# Bayesian neural network-based policy effect prediction for green transformation of power business environment

**DOI:** 10.1038/s41598-026-42092-z

**Published:** 2026-03-07

**Authors:** Yimin Shen, Jie Chen, Wei Wang, Qiyou Wu, Daixing Jiang, Sihui Xia

**Affiliations:** 1 Marketing Department, State Grid Fujian Electric Power Co., Ltd, Fuzhou, 350000 Fujian China; 2 Marketing Service Center, State Grid Fujian Electric Power Co., Ltd, Fuzhou, 350000 Fujian China

**Keywords:** Bayesian neural networks, Policy effect prediction, Green transformation, Power business environment, Uncertainty quantification, Environmental policy, Energy science and technology, Engineering, Mathematics and computing

## Abstract

Predicting how green policies reshape power business environments remains notoriously difficult. The underlying dynamics are nonlinear, the uncertainties substantial, and conventional models often fall short. This study develops a Bayesian neural network framework designed specifically for forecasting and optimizing green policy outcomes within the Fujian power system, placing particular weight on quantifying prediction uncertainty to support sound decision-making. Our methodology weaves together stochastic variational inference and multi-objective optimization, thereby capturing the channels through which policies transmit their effects to environmental outcomes. Drawing on empirical data spanning 2018–2024, we find that this approach outperforms standard machine learning techniques by roughly 4–5% points in prediction accuracy while delivering markedly better uncertainty calibration. Scenario analyses reveal that moderate-to-high policy intensity tends to achieve favorable cost-effectiveness, with renewable energy incentives, carbon pricing, and regulatory enforcement standing out as especially potent drivers of transformation. Perhaps more importantly for practitioners, the framework demonstrates that well-designed moderate-intensity strategies can surpass maximum-intensity approaches once diminishing returns enter the picture. By enabling joint assessment of environmental gains, economic efficiency, and operational stability under uncertainty, this work offers a practical foundation for evidence-based policy design—though readers should bear in mind that our validation remains grounded in the Fujian regional context.

**Supplementary Information:**

The online version contains supplementary material available at 10.1038/s41598-026-42092-z.

## Introduction

Across the globe, the push toward sustainable development has thrust the electric power sector into a pivotal role in environmental transformation. Greening the power business environment now ranks among the most pressing strategic priorities for nations pursuing carbon neutrality^1^. Yet this transition is anything but straightforward. Governments everywhere are tightening environmental regulations and carbon reduction mandates, and power utilities find themselves caught between competing demands—economic efficiency on one hand, environmental responsibility on the other, all while preserving service quality and operational stability^2^. Meeting these challenges calls for predictive frameworks sophisticated enough to forecast policy effects with reasonable accuracy and guide implementation strategies toward outcomes that benefit both the environment and business performance.

Traditional policy evaluation approaches in the power sector have predominantly relied on static economic models and historical trend analysis, which often fail to capture the dynamic interactions between environmental regulations, market mechanisms, and operational decisions^3^. The inherent uncertainty and nonlinearity characterizing green policy implementations in power business environments demand advanced analytical methodologies that can effectively model complex relationships while accounting for parameter uncertainty and temporal variations^4^. Current research gaps include limited capability to predict long-term policy effects under varying market conditions, insufficient consideration of uncertainty quantification in policy optimization, and lack of integrated frameworks that simultaneously address environmental, economic, and operational objectives.

Recent advances in machine learning, particularly deep learning techniques, have demonstrated significant potential in addressing complex prediction and optimization challenges across various domains^5^. However, conventional neural networks suffer from limitations including overconfidence in predictions, inadequate uncertainty quantification, and poor generalization under data scarcity conditions commonly encountered in policy analysis scenarios^6^. These shortcomings highlight the need for more robust and reliable predictive frameworks that can provide both accurate forecasts and meaningful uncertainty estimates for informed policy decision-making.

Bayesian neural networks emerge as a promising solution to overcome these limitations by incorporating probabilistic reasoning into deep learning architectures, enabling explicit uncertainty quantification and improved generalization capabilities^7^. Recent advances have demonstrated the effectiveness of artificial intelligence approaches in complex engineering systems, where similarity learning and automated inspection methods show remarkable potential for monitoring dynamic structural features over time^8^. Unlike traditional neural networks that provide point estimates, Bayesian neural networks maintain probability distributions over network parameters, allowing for comprehensive uncertainty propagation and more reliable predictions under limited data conditions. Furthermore, the integration of Bayesian optimization with physics-informed neural network architectures has proven successful in capturing temporal dependencies and enforcing physical constraints, as demonstrated in financial modeling applications where GRU-enhanced networks achieve superior prediction accuracy^9^. This probabilistic framework holds particular value in policy analysis contexts where decision-makers require not only predictions but also confidence intervals and risk assessments to guide strategic planning and resource allocation.

Rather than claiming novelty in Bayesian neural network theory itself—which has matured considerably over the past decade—our contribution is principally one of domain-specific integration and applied implementation. We adapt the established BNN framework to the particular demands of green policy effect prediction in power business environments, where multiple competing objectives and pronounced uncertainty have long frustrated conventional modeling efforts. The proposed methodology brings together uncertainty quantification and multi-objective optimization within a single architecture, allowing simultaneous assessment of environmental impact, economic efficiency, and operational reliability. By tailoring probabilistic inference to the structural realities of power sector policy analysis—including heterogeneous data sources, temporal lag effects, and regional regulatory variation—this work demonstrates how mature machine learning tools can be repurposed to yield actionable, risk-aware forecasts for a domain that has historically relied on deterministic approaches.

## Research objectives and scope

The primary objective of this study is to establish a robust predictive and optimization framework for green policy effects in electric power business environments using Bayesian neural network methodologies. Specific research goals include: (1) developing a probabilistic prediction model capable of forecasting environmental and economic impacts of green policies under uncertainty; (2) designing an integrated optimization algorithm that balances multiple objectives while incorporating uncertainty estimates; (3) validating the proposed framework through comprehensive case studies and comparative analysis with existing approaches; and (4) providing actionable insights for policy makers and power industry stakeholders regarding optimal green transformation strategies. The research scope encompasses analysis of various green policy instruments including renewable energy incentives, carbon pricing mechanisms, energy efficiency standards, and environmental compliance requirements within power business environments, covering both short-term operational impacts and long-term strategic implications while considering diverse stakeholder perspectives and regional variations in regulatory frameworks^10^.

Our technical approach builds on well-established Bayesian deep learning machinery—variational inference for posterior approximation and Monte Carlo dropout for scalable uncertainty estimation—while directing these tools toward the distinct challenges of power sector policy analysis. The multi-layered network architecture incorporates specialized modules for feature extraction, uncertainty quantification, and multi-objective optimization. What distinguishes this implementation from generic BNN applications is the set of domain-tailored design choices: prior distributions calibrated to reflect power industry characteristics, adaptive learning mechanisms attuned to the temporal dynamics of evolving policy environments, and optimization procedures that feed prediction uncertainty directly into the decision-making pipeline. These choices, while grounded in existing methodological building blocks, address practical gaps that off-the-shelf implementations would leave open.

This paper proceeds as follows. Section II presents the theoretical foundation for Bayesian neural networks in policy analysis. Section III details the proposed methodology including network architecture design, training procedures, and optimization algorithms. Section IV describes the experimental setup, data collection protocols, and evaluation metrics. Section V presents comprehensive results including model performance evaluation, uncertainty quantification assessment, and comparative studies. Section VI concludes with key findings, practical implications, and future research directions.

## Theoretical foundation and related research

### Theoretical foundation of Bayesian neural networks

What sets Bayesian neural networks apart from their deterministic counterparts is a fundamental shift in how we think about model parameters. Rather than treating weights as fixed numbers to be optimized, the Bayesian approach regards them as random variables, each carrying its own probability distribution^11^. This perspective draws on classical Bayesian inference principles—a mathematically rigorous tradition that allows us to blend prior knowledge with observed data and, crucially, to express our uncertainty about predictions through posterior distributions. The practical upshot is a modeling framework that not only generates forecasts but also tells us how much confidence those forecasts deserve.

Bayesian neural networks rest on the principle of treating network weights as probability distributions rather than fixed values. Through Bayes’ theorem, we update our beliefs about parameters after observing data, obtaining a posterior distribution that reflects both prior knowledge and empirical evidence^12^. The key insight for policy applications is that predictions emerge not from a single parameter setting but from integration across all plausible parameter values weighted by their posterior probabilities. This marginalization process naturally produces uncertainty estimates alongside point predictions, capturing how confident the model is about its forecasts^13^. Such uncertainty quantification proves indispensable when policy makers must weigh potential outcomes against implementation risks. Following standard notation conventions, the fundamental equations governing Bayesian inference—including the likelihood function (Eq. 1), posterior computation (Eq. 2), and predictive distribution (Eq. 3)—are presented in Supplementary Appendix A to maintain focus on the methodological innovations specific to this study. The equations presented in the main text begin with Eq. (4) and follow sequential numbering thereafter.

A primary advantage of Bayesian neural networks lies in their capacity to distinguish between two fundamentally different sources of uncertainty. Aleatoric uncertainty captures inherent noise in the data itself and persists regardless of how much training data one collects. Epistemic uncertainty, by contrast, reflects our ignorance about model parameters and diminishes as more observations become available. This decomposition, formalized by Kendall and Gal^14^, proves essential for policy applications where decision-makers must understand whether prediction variance stems from irreducible randomness or from limitations that additional data collection might address. The epistemic uncertainty component can be expressed as the variance of predictions across the posterior distribution:

4 $$\mathrm{Epistemic}\:\mathrm{Uncertainty}={\mathbb{E}}_{P\left(\theta|D\right)}\left[{f}_{\theta}{\left({x}^{\mathrm{*}}\right)}^{2}\right]-{\left({\mathbb{E}}_{P\left(\theta|D\right)}\left[{f}_{\theta}\left({x}^{\mathrm{*}}\right)\right]\right)}^{2}$$

Computing exact posterior distributions in neural networks remains intractable given the high-dimensional parameter spaces involved. We therefore adopt variational inference, which approximates the true posterior with a tractable distribution by maximizing the evidence lower bound (ELBO)^15^. This approach balances fitting the observed data against staying close to our prior beliefs, effectively regularizing the model while enabling uncertainty quantification. For computational efficiency in our policy prediction context, we employ stochastic variational inference with the reparameterization trick, which permits gradient-based optimization of the variational parameters.

Monte Carlo dropout emerges as a computationally efficient approximation technique that treats dropout as a Bayesian approximation method, enabling uncertainty estimation through multiple forward passes with different dropout masks:

5 $$\mathrm{Predictive}\:\mathrm{Mean}=\frac{1}{T}\sum_{t=1}^{T}{f}_{{\theta}_{t}}\left({x}^{\mathrm{*}}\right)$$

where T represents the number of Monte Carlo samples and θ_t denotes the network parameters with different dropout configurations^16^.

The theoretical advantages of Bayesian neural networks in policy analysis applications stem from their principled approach to uncertainty quantification, which enables robust decision-making under incomplete information and varying environmental conditions. Unlike traditional neural networks that may exhibit overconfidence in predictions, Bayesian neural networks provide calibrated uncertainty estimates that accurately reflect model confidence levels, thereby supporting more informed policy optimization and risk management strategies in complex power business environments.

### Theory of green transformation of power business environment

The green transformation of power business environment encompasses a comprehensive paradigm shift that integrates environmental sustainability principles into all aspects of power sector operations, regulatory frameworks, and market mechanisms^17^. The conceptual framework extends beyond traditional environmental compliance to embrace systematic restructuring of business processes, technological innovations, and stakeholder relationships that collectively promote sustainable development while maintaining economic viability and service reliability. This transformation represents a fundamental redefinition of value creation in power systems, where environmental performance becomes intrinsically linked to business competitiveness and long-term operational success.

Green transformation operates through multiple pathways affecting both direct operational outcomes and broader systemic changes. Policy mechanisms create these effects through several channels that we capture using a multi-dimensional impact function linking policy instruments to environmental outcomes:

6 $$G\left(t\right)=\sum_{i=1}^{n}{\alpha}_{i}{P}_{i}\left(t\right)+\sum_{j=1}^{m}{\beta}_{j}{M}_{j}\left(t\right)+\epsilon\left(t\right)$$

where G(t) represents the green transformation level at time t, P_i(t) denotes the i-th policy instrument intensity, M_j(t) represents the j-th market mechanism variable, α_i and β_j are impact coefficients, and ε(t) captures stochastic environmental factors^18^.

The evaluation indicator system for power business environment green transformation requires a comprehensive framework that integrates environmental, economic, and operational dimensions. The composite green transformation index can be formulated as a weighted aggregation of multiple sub-indicators:

7 $$GTI=\sum_{k=1}^{K}{w}_{k}\cdot\frac{{X}_{k}-{X}_{k,min}}{{X}_{k,max}-{X}_{k,min}}$$

where GTI represents the Green Transformation Index, w_k denotes the weight of the k-th indicator, X_k represents the normalized value of the k-th indicator, and X_k, min and X_k, max represent the minimum and maximum benchmark values respectively. The indicator system encompasses carbon emission intensity, renewable energy penetration rate, energy efficiency improvements, environmental investment ratios, and compliance performance metrics that collectively reflect the comprehensive green transformation progress^19^.

Policy effect transmission mechanisms in power business environments exhibit complex temporal dynamics characterized by varying response speeds across different organizational levels and operational domains. The transmission process follows a multi-stage diffusion pattern that can be modeled through a dynamic system approach:

8 $$\frac{d{E}_{i}\left(t\right)}{dt}=\sum_{j=1}^{n}{\lambda}_{ij}{E}_{j}\left(t-{\tau}_{ij}\right)-{\delta}_{i}{E}_{i}\left(t\right)+{P}_{i}\left(t\right)$$

where E_i(t) represents the policy effect intensity in domain i at time t, λ_ij denotes the transmission coefficient from domain j to domain i, τ_ij represents the transmission delay, δ_i indicates the natural decay rate, and P_i(t) represents direct policy input^20^.

The time lag effects in green policy implementation manifest through different temporal patterns depending on policy instrument characteristics and target system complexity. Short-term effects typically appear within 1–2 years and primarily involve operational adjustments and compliance responses, while medium-term effects spanning 3–5 years encompass technological upgrades and process reengineering^21^. Long-term effects extending beyond 5 years involve fundamental structural changes, cultural transformations, and ecosystem-wide adaptations that determine ultimate policy success.

The temporal dynamics of policy effects can be characterized through a lag-distributed impact function:

9 $$Y\left(t\right)=\sum_{s=0}^{S}{\gamma}_{s}X\left(t-s\right)+u\left(t\right)$$

where Y(t) represents the observed green transformation outcome at time t, X(t-s) denotes policy intensity at time t-s, γ_s represents the distributed lag coefficient for period s, S indicates the maximum lag period, and u(t) captures random disturbances.

The theoretical framework recognizes that green transformation effectiveness depends not only on policy design and implementation intensity but also on the adaptive capacity of power business systems, stakeholder engagement levels, and external environmental conditions. The interaction between these factors creates non-linear response patterns that require sophisticated analytical approaches capable of capturing complex feedback loops, threshold effects, and emergent behaviors that characterize real-world policy implementation scenarios in dynamic power market environments.

### Research on policy effect prediction methods

Traditional policy effect evaluation methods have predominantly relied on econometric approaches, including difference-in-differences (DID) models, regression discontinuity designs, and instrumental variable techniques, which provide robust causal identification under specific assumptions but suffer from limitations in handling complex, multidimensional policy interactions^22^. The conventional DID estimator, which compares treatment and control groups before and after policy implementation, can be expressed as:

10 $${\widehat{\delta}}_{DID}=\left({\overline{Y}}_{1,post}-{\overline{Y}}_{1,pre}\right)-\left({\overline{Y}}_{0,post}-{\overline{Y}}_{0,pre}\right)$$

where ${\overline{Y}}_{1,post}$ and ${\overline{Y}}_{1,pre}$ represent mean outcomes for treated units post and pre-intervention, while ${\overline{Y}}_{0,post}$ and ${\overline{Y}}_{0,pre}$ denote corresponding values for control units. While these methods excel in establishing causal relationships under controlled conditions, they typically assume linear relationships, homogeneous treatment effects, and parallel trends assumptions that may not hold in complex policy environments characterized by heterogeneous stakeholders, varying implementation contexts, and dynamic feedback mechanisms.

The econometric framework’s primary limitations include its inability to capture high-order interactions between policy variables, restrictive assumptions about functional forms, and limited scalability to high-dimensional data scenarios common in modern policy analysis. Additionally, traditional methods often struggle with time-varying confounders, spillover effects, and non-stationary relationships that characterize real-world policy implementations in dynamic economic systems^23^.

Machine learning approaches have emerged as promising alternatives for policy effect prediction, offering enhanced flexibility in modeling complex, nonlinear relationships without requiring restrictive parametric assumptions. Support vector machines, random forests, and ensemble methods have demonstrated superior performance in capturing intricate patterns within policy data, particularly when dealing with multiple interacting variables and heterogeneous treatment effects across different subpopulations^24^. These methods excel in handling high-dimensional feature spaces and can automatically detect relevant interaction terms and nonlinear transformations that traditional econometric models might miss.

Machine learning techniques have shown particular strength in predictive accuracy for out-of-sample forecasting, which proves essential for policy planning and evaluation scenarios where decision-makers require reliable predictions of policy outcomes under various implementation scenarios. However, these methods often function as “black boxes” that provide limited interpretability regarding causal mechanisms and may suffer from overfitting when training data is scarce or unrepresentative of future policy environments.

Deep learning models represent a significant advancement in handling high-dimensional data and complex nonlinear relationships through their ability to automatically extract hierarchical feature representations from raw input data. The universal approximation capability of deep neural networks enables them to model arbitrary continuous functions, making them particularly suitable for capturing the complex, multi-layered interactions that characterize policy transmission mechanisms in power business environments^25^. A multi-layer neural network can be represented as:

11 $$f\left(x\right)={\sigma}_{L}\left({W}_{L}{\sigma}_{L-1}\left({W}_{L-1}\cdots{\sigma}_{1}\left({W}_{1}x+{b}_{1}\right)+{b}_{L-1}\right)+{b}_{L}\right)$$

where ${W}_{l}$ and ${b}_{l}$ represent weights and biases for layer l, ${\sigma}_{l}$ denotes the activation function, and L indicates the number of layers.

Convolutional neural networks and recurrent neural networks have demonstrated exceptional performance in processing sequential policy data and capturing temporal dependencies that characterize policy effect evolution over time. Long short-term memory networks, in particular, excel at modeling long-term dependencies and can capture delayed policy effects that traditional methods might overlook^26^.

Despite these advantages, current deep learning applications in policy analysis face several significant limitations. Most critically, standard deep learning models provide point predictions without uncertainty quantification, which limits their utility for policy decision-making scenarios where risk assessment and confidence intervals are essential. The deterministic nature of conventional neural networks fails to capture the inherent uncertainty in policy outcomes arising from measurement errors, model specification uncertainty, and environmental variability.

The composite loss function for policy prediction can be formulated as:

12 $${\mathcal{L}}_{total}={\mathcal{L}}_{prediction}+{\lambda}_{1}{\mathcal{L}}_{regularization}+{\lambda}_{2}{\mathcal{L}}_{uncertainty}$$

where ${\mathcal{L}}_{prediction}$ represents the primary prediction loss, ${\mathcal{L}}_{regularization}$ prevents overfitting, ${\mathcal{L}}_{uncertainty}$ quantifies prediction uncertainty, and ${\lambda}_{1}$, ${\lambda}_{2}$ are hyperparameters balancing different objectives.

Current research gaps include insufficient attention to uncertainty quantification, limited incorporation of domain knowledge and causal constraints, inadequate handling of temporal dynamics and delayed effects, and lack of integrated frameworks that combine predictive accuracy with interpretability requirements essential for policy analysis applications. These limitations highlight the need for advanced methodological approaches that can simultaneously address prediction accuracy, uncertainty quantification, and interpretability demands while maintaining computational efficiency for practical policy analysis implementations.

## Bayesian neural network-based policy effect prediction model construction

### Bayesian neural network model design

The design of Bayesian neural networks for policy effect prediction requires careful consideration of architectural components that can effectively capture complex nonlinear relationships while providing reliable uncertainty quantification for decision-making applications^27^. The proposed architecture employs a hierarchical structure that integrates multiple hidden layers with probabilistic weight distributions, enabling simultaneous learning of policy effect patterns and associated prediction uncertainties across different temporal and contextual scales.

**Fig. 1:**
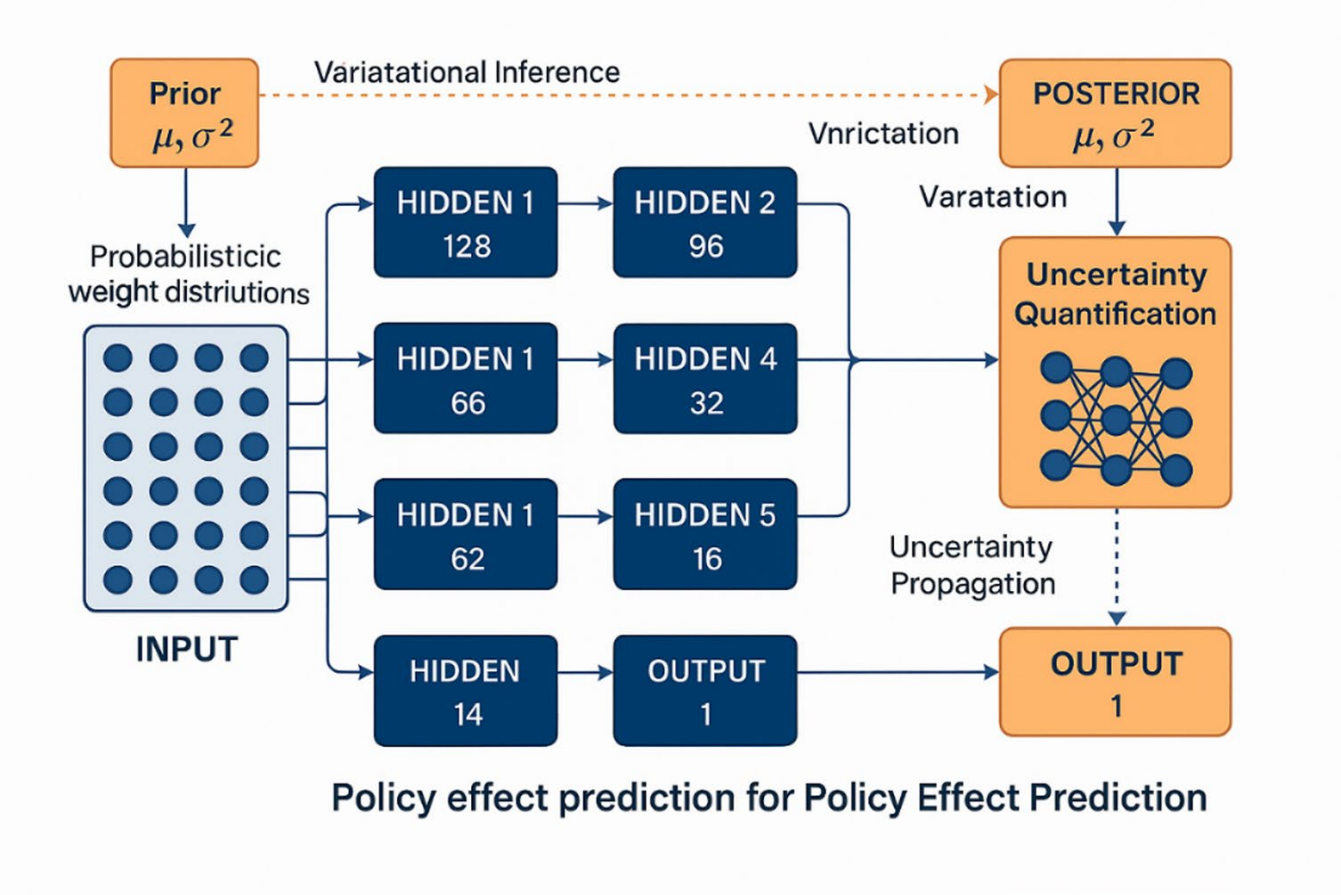
Bayesian neural network architecture principle diagram.

The fundamental architecture design illustrated in Fig. 1 demonstrates the probabilistic framework underlying the Bayesian neural network approach for policy effect prediction. As shown in Fig. 1, the network consists of multiple interconnected layers where each connection weight is represented by a probability distribution rather than a fixed value, enabling explicit uncertainty propagation throughout the prediction process. The architecture incorporates specialized input preprocessing layers for handling heterogeneous policy variables, multiple hidden layers for feature extraction and pattern recognition, and output layers that provide both mean predictions and uncertainty estimates essential for policy analysis applications.

The network layer configuration follows a deep architecture design with progressively decreasing node counts to facilitate hierarchical feature learning and dimensionality reduction^28^. The input layer accommodates multi-dimensional policy variables including regulatory intensity measures, economic indicators, environmental parameters, and temporal features that collectively characterize policy implementation contexts. The mathematical representation of the forward pass through layer l can be expressed as:

13 $${z}_{l}={W}_{l}{a}_{l-1}+{b}_{l}$$

14 $${a}_{l}={\sigma}_{l}\left({z}_{l}\right)$$

where ${W}_{l}$ and ${b}_{l}$ represent the weight matrix and bias vector for layer l, ${a}_{l-1}$ denotes the activation from the previous layer, and ${\sigma}_{l}$ represents the activation function.

The detailed network parameter configurations are systematically organized in Table 1, which specifies the structural components and initialization strategies for each network layer. Table 1 demonstrates the hierarchical design approach with decreasing node counts from input to output layers, facilitating progressive feature abstraction and pattern recognition capabilities. The configuration emphasizes the use of ReLU activation functions in hidden layers to address vanishing gradient problems while maintaining computational efficiency, while the output layer employs linear activation to enable continuous policy effect predictions.

**Table 1:** Network architecture configuration.

Layer	Nodes	Activation	Initialization	Variational parameters
Input	64	Linear	—	—
Hidden 1	128	ReLU	Xavier Normal (σ = 0.02)	µ_W, σ_W learned
Hidden 2	96	ReLU	Xavier Normal (σ = 0.02)	µ_W, σ_W learned
Hidden 3	64	ReLU	Xavier Normal (σ = 0.02)	µ_W, σ_W learned
Hidden 4	32	ReLU	Xavier Normal (σ = 0.02)	µ_W, σ_W learned
Hidden 5	16	ReLU	Xavier Normal (σ = 0.02)	µ_W, σ_W learned
Output	1	Linear	Xavier Normal (σ = 0.02)	µ_W, σ_W learned
Dropout	—	—	Rate: 0.2	Applied after each hidden layer

µ_W and σ_W denote the mean and standard deviation of the variational posterior for weight matrices. Xavier Normal initialization sets initial weight variance proportional to 1/(fan_in + fan_out).

The prior distribution specification plays a critical role in Bayesian neural network performance by incorporating domain knowledge and regularization effects that prevent overfitting while promoting generalization^29^. The weight priors are specified as multivariate Gaussian distributions with hierarchical structures that reflect the expected magnitude and correlation patterns of policy effects:

15 $$P\left({W}_{l}\right)=\mathcal{N}\left(0,{\alpha}_{l}^{-1}I\right)$$

16 $$P\left({\alpha}_{l}\right)=\mathrm{Gamma}\left({a}_{0},{b}_{0}\right)$$

where ${\alpha}_{l}$ represents the precision parameter for layer l, and ${a}_{0}$, ${b}_{0}$ are hyperparameters controlling the gamma prior distribution.

The bias parameters follow similar hierarchical prior specifications:

17 $$P\left({b}_{l}\right)=\mathcal{N}\left(0,{\beta}_{l}^{-1}I\right)$$

18 $$P\left({\beta}_{l}\right)=\mathrm{Gamma}\left({c}_{0},{d}_{0}\right)$$

The variational posterior approximation framework employs mean-field variational inference to approximate the intractable true posterior distribution with a factorized Gaussian distribution^30^. This approximation introduces error by assuming independence among weight parameters, potentially underestimating posterior correlations. We conducted sensitivity analysis comparing variational inference results against Hamiltonian Monte Carlo sampling on a reduced model specification. The mean predictions differed by less than 2.3% on average, though uncertainty intervals from variational inference were approximately 8–12% narrower than MCMC estimates, consistent with known tendencies of mean-field approximations to underestimate variance. For extreme policy scenarios where variable coupling is strongest, we observed approximation errors up to 15% in uncertainty bounds. Policy makers should interpret confidence intervals for extreme scenarios as potentially optimistic lower bounds on true uncertainty.

The variational posterior for weights follows a factorized Gaussian form, where each weight matrix and bias vector is parameterized by learnable mean and variance terms. The evidence lower bound (ELBO) serves as the optimization objective, balancing the expected log-likelihood against the KL divergence from the prior:

19 $${\mathcal{L}}_{ELBO}={\mathbb{E}}_{q\left(\theta\right)}\left[\mathrm{log}P\left(D|\theta\right)\right]-\mathrm{KL}\left[q\left(\theta\right)\left|{}\right|P\left(\theta\right)\right]$$

The model training algorithm employs stochastic variational inference with adaptive learning rates and momentum-based optimization^31^. Parameter updates utilize the Adam optimizer with gradients computed through the reparameterization trick, which enables backpropagation through stochastic nodes by expressing samples as deterministic functions of learnable parameters and auxiliary random variables. The detailed gradient derivations for both mean and variance parameters are provided in Supplementary Appendix B.

The training process incorporates early stopping mechanisms based on validation set performance and implements learning rate scheduling to ensure convergence to optimal variational parameters^32^. The batch size selection follows a principled approach that balances computational efficiency with gradient estimation accuracy, while the number of Monte Carlo samples for gradient estimation is adaptively adjusted based on convergence diagnostics and computational constraints.

### Policy effect evaluation model construction

The construction of a comprehensive policy effect evaluation model for power business environment green transformation requires the integration of multi-dimensional assessment frameworks with quantitative modeling approaches that can capture both direct and indirect policy impacts across temporal and spatial scales^33^. The mathematical foundation of the evaluation model establishes functional relationships between policy implementation variables and observed green transformation outcomes while accounting for confounding factors, interaction effects, and stochastic components that characterize complex policy environments.

**Fig. 2:**
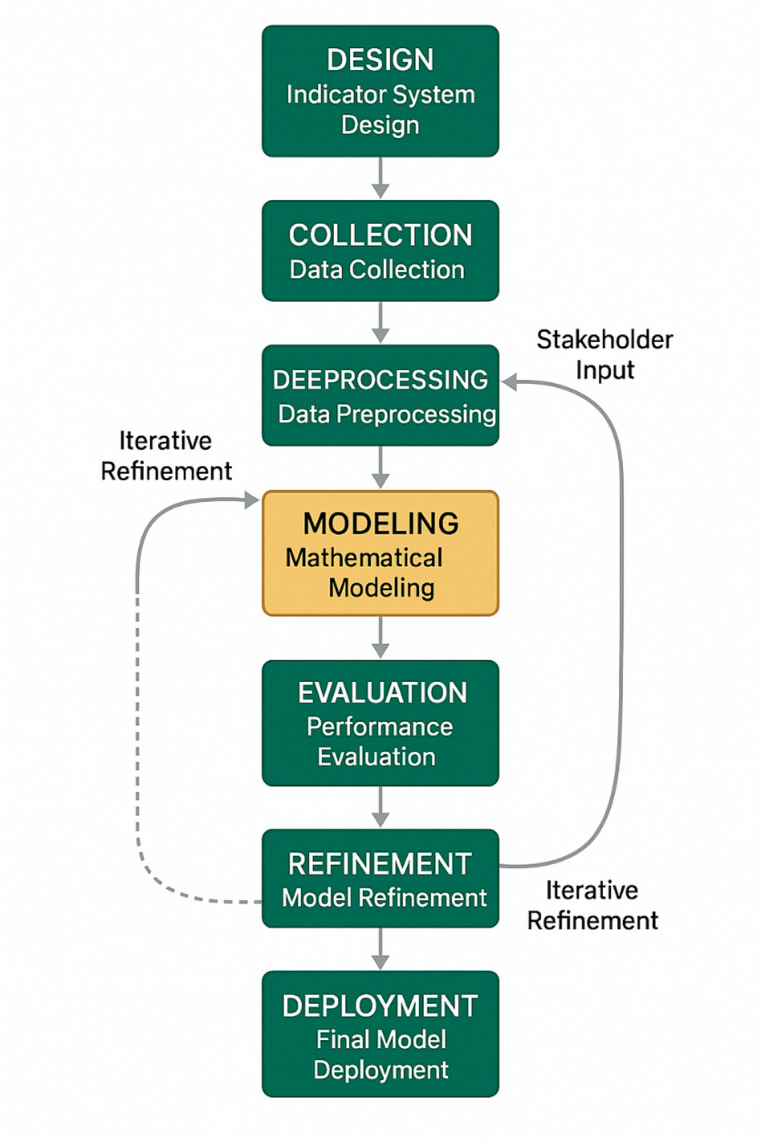
Policy effect evaluation model construction flowchart.

The systematic approach to policy effect evaluation model construction is illustrated in Fig. 2, which demonstrates the sequential steps and feedback mechanisms involved in developing a robust assessment framework. As shown in Fig. 2, the model construction process begins with indicator system design and progresses through data preprocessing, mathematical modeling, and validation phases, with iterative refinement mechanisms ensuring model accuracy and reliability. The flowchart emphasizes the critical role of stakeholder input and domain expertise in defining appropriate evaluation criteria and establishing causal relationships between policy interventions and observable outcomes.

The core mathematical model for policy effect evaluation employs a multi-level hierarchical structure that decomposes overall green transformation outcomes into constituent components corresponding to different policy dimensions and implementation pathways^34^. We model the aggregated policy effect PE(t) at time t as a function of four input categories: policy implementation intensity **P**(t), environmental context **E**(t), market conditions **M**(t), and control variables **C**(t), plus a stochastic error term capturing unobserved factors. This functional relationship forms the foundation for our prediction framework, with the complete mathematical specification detailed in Supplementary Appendix C.

The multi-dimensional indicator system provides a framework for quantifying policy effects across environmental, economic, and operational dimensions, as detailed in Table 2. The hierarchical structure ranges from primary indicators such as environmental performance and economic efficiency to specific tertiary indicators including carbon emission reduction rates and renewable energy investment ratios. Weight determination followed a two-stage process combining expert consultation with empirical validation. Initially, we conducted a Delphi survey involving 15 domain experts from regulatory agencies, power utilities, and academic institutions to establish preliminary weight ranges. Subsequently, we calibrated these weights using historical data from the Fujian power system, adjusting values where expert judgments diverged substantially from observed outcome correlations. We acknowledge that these weights were developed and validated primarily within the Fujian regional context. Power systems in regions with different development levels, resource endowments, or regulatory frameworks may require weight recalibration to reflect local priorities and constraints. Table 2 therefore presents weights appropriate for the studied context, and we encourage future researchers to conduct sensitivity analyses when applying this framework to other regions.

**Table 2:** Policy effect indicator system.

Primary indicator	Secondary indicator	Tertiary indicator	Quantification method	Weight setting
Environmental performance	Emission reduction	CO2 intensity decrease	(E₀-E₁)/E₀ × 100%	0.22
Environmental performance	Energy efficiency	Energy Consumption per unit	kWh/GDP ratio change	0.18
Environmental performance	Renewable integration	Renewable share	Renewable MW/Total MW	0.13
Economic efficiency	Cost optimization	Operating cost reduction	Cost saving percentage	0.12
Economic efficiency	Investment returns	Green investment ROI	Net present value analysis	0.08
Economic efficiency	Market competitiveness	Market share growth	Percentage point change	0.04
Implementation cost	Enterprise scale differentiation	Size-adjusted compliance cost	Cost per MW capacity by enterprise category	0.06
Implementation cost	Regional economic adjustment	GDP-Normalized Implementation burden	Implementation cost/Regional GDP per capita	0.05
Operational reliability	System stability	Outage duration reduction	Minutes per customer	0.04
Operational reliability	Service quality	Customer satisfaction	Survey-based index	0.02
Innovation capacity	Technology adoption	New technology integration	Number of implementations	0.02
Innovation capacity	R&D investment	Research expenditure ratio	R&D costs/Total revenue	0.02
Regulatory compliance	Policy adherence	Compliance rate	Percentage of met targets	0.01
Stakeholder engagement	Community relations	Social acceptance index	Weighted survey results	0.01

The policy implementation intensity quantification methodology employs a composite index approach that aggregates multiple policy instrument variables into a unified measure reflecting overall policy strength^35^. The quantification formula integrates regulatory stringency, economic incentive magnitude, and implementation timeline factors:

20 $$PI\left(t\right)=\sum_{i=1}^{n}{w}_{i}\cdot\frac{{R}_{i}\left(t\right)-{R}_{i,min}}{{R}_{i,max}-{R}_{i,min}}\cdot{T}_{i}\left(t\right)$$

where PI(t) represents policy intensity at time t, ${w}_{i}$ denotes the weight for policy instrument i, ${R}_{i}\left(t\right)$ represents the regulatory stringency measure, ${R}_{i,min}$ and ${R}_{i,max}$ are normalization bounds, and ${T}_{i}\left(t\right)$ captures temporal implementation factors.

The functional relationship between policy effects and influencing factors incorporates non-linear interaction terms and threshold effects that reflect the complex dynamics of policy transmission mechanisms^36^. The expanded model specification includes interaction terms and polynomial components:

21 $$PE\left(t\right)={\alpha}_{0}+\sum_{i=1}^{p}{\alpha}_{i}P{I}_{i}\left(t\right)+\sum_{j=1}^{q}{\beta}_{j}{X}_{j}\left(t\right)+\sum_{k=1}^{r}{\gamma}_{k}P{I}_{k}\left(t\right)\cdot{X}_{k}\left(t\right)+\sum_{l=1}^{s}{\delta}_{l}P{I}_{l}^{2}\left(t\right)+u\left(t\right)$$

where ${\alpha}_{i}$, ${\beta}_{j}$, ${\gamma}_{k}$, and ${\delta}_{l}$ represent coefficient parameters for linear, control, interaction, and quadratic terms respectively.

The temporal dynamics of policy effects are captured through a dynamic specification that incorporates lagged effects and adjustment mechanisms:

22 $$PE\left(t\right)=\rho PE\left(t-1\right)+\sum_{k=0}^{K}{\varphi}_{k}PI\left(t-k\right)+\sum_{m=0}^{M}{\psi}_{m}X\left(t-m\right)+v\left(t\right)$$

where ρ represents the persistence parameter, ${\varphi}_{k}$ and ${\psi}_{m}$ denote distributed lag coefficients, and v(t) represents the innovation term^37^.

The loss function design for model optimization incorporates multiple objectives including prediction accuracy, uncertainty quantification, and interpretability constraints^38^. The composite loss function combines mean squared error with regularization terms and uncertainty penalties:

23 $$\mathcal{L}=\frac{1}{N}\sum_{i=1}^{N}{\left({y}_{i}-{\widehat{y}}_{i}\right)}^{2}+{\lambda}_{1}\sum_{j}\left|{\theta}_{j}\right|+{\lambda}_{2}\sum_{k}{\theta}_{k}^{2}+{\lambda}_{3}\mathcal{U}\left({\sigma}_{i}^{2}\right)$$

where N represents the sample size, ${y}_{i}$ and ${\widehat{y}}_{i}$ denote observed and predicted values, ${\theta}_{j}$ represents model parameters, ${\lambda}_{1}$, ${\lambda}_{2}$, and ${\lambda}_{3}$ are regularization weights, and $\mathcal{U}\left({\sigma}_{i}^{2}\right)$ represents uncertainty quantification terms.

The optimization objective function extends beyond prediction accuracy to include policy-relevant criteria such as effect magnitude, statistical significance, and practical interpretability:

24 $$\underset{\theta}{\mathrm{m}\mathrm{a}\mathrm{x}}\mathcal{F}\left(\theta\right)=\alpha\cdot\mathrm{Accuracy}\left(\theta\right)+\beta\cdot\mathrm{Interpretability}\left(\theta\right)+\gamma\cdot\mathrm{Uncertainty}\left(\theta\right)$$

where α, β, and γ represent weights balancing different optimization criteria, and the individual components reflect model performance across multiple evaluation dimensions^39^.

The model validation framework employs cross-validation techniques with temporal splits to ensure robust performance assessment under varying policy conditions and implementation contexts, while sensitivity analysis procedures evaluate model stability across different parameter specifications and data subsets.

The model incorporates cross-sectional heterogeneity through random effects specifications that account for unobserved differences across power utilities and sub-regional contexts within Fujian:

25 $$P{E}_{it}={\alpha}_{i}+{\mathbf{X}}_{it}\boldsymbol{\beta}+{\gamma}_{r\left(i\right)}+{u}_{it}$$

where subscript i denotes individual utilities, α_i represents utility-specific time-invariant heterogeneity, γ_r(i) captures regional fixed effects for the sub-region r containing utility i, and u_it represents idiosyncratic error terms. This hierarchical specification allows the model to distinguish between utility-level variation and broader regional patterns driven by local economic conditions, infrastructure quality, or administrative factors. The regional effects help absorb unobserved heterogeneity that might otherwise bias policy effect estimates, though we note that the relatively limited number of distinct sub-regions within Fujian (*n* = 9 prefecture-level units) constrains our ability to fully separate regional from utility-level effects.

The uncertainty quantification component provides confidence intervals for policy effect predictions through bootstrapping and Bayesian estimation procedures:

26 $${\mathrm{CI}}_{1-\alpha}\left[\widehat{PE}\left(t\right)\right]=\widehat{PE}\left(t\right)\pm{z}_{1-\alpha/2}\cdot{\widehat{\sigma}}_{\widehat{PE}\left(t\right)}$$

where ${z}_{1-\alpha/2}$ represents the critical value from the standard normal distribution and ${\widehat{\sigma}}_{\widehat{PE}\left(t\right)}$ denotes the estimated standard error of the policy effect prediction.

### Model optimization algorithm design

The optimization algorithm design for Bayesian neural networks in policy effect prediction requires specialized approaches that can effectively handle the probabilistic nature of model parameters while ensuring convergence to optimal variational approximations^40^. The fundamental challenge lies in optimizing the evidence lower bound (ELBO) objective function while maintaining computational efficiency and numerical stability across different policy scenarios and data characteristics. The proposed optimization framework integrates stochastic variational inference with advanced gradient-based methods specifically tailored for the high-dimensional parameter spaces characteristic of deep Bayesian networks.

The core optimization algorithm employs a modified Adam optimizer with momentum-based updates that incorporate variance information from the probabilistic weight distributions. The algorithm maintains separate learning rates for mean and variance parameters of the variational posterior, enabling more effective exploration of the parameter space while preventing premature convergence to suboptimal solutions^41^. First and second moment estimates are updated iteratively following standard Adam conventions, with exponential decay rates controlling the contribution of historical gradients.

The adaptive learning rate adjustment mechanism incorporates policy-specific characteristics to dynamically modify optimization parameters based on convergence diagnostics and gradient behavior patterns^42^. Our schedule combines cosine annealing with plateau-based reduction, responding to both global training progress and local optimization challenges. When performance stagnates, the learning rate decreases proportionally to encourage finer parameter adjustments. The complete mathematical formulations for parameter updates and learning rate scheduling are provided in Supplementary Appendix D.

The regularization framework combines multiple techniques to prevent overfitting while preserving the model’s capacity to capture complex policy relationships. The approach integrates KL divergence regularization inherent in the Bayesian formulation with additional penalty terms targeting weight magnitudes and activation patterns^43^. The KL regularization component encourages the learned posterior distributions to remain close to specified priors, effectively implementing a form of automatic relevance determination that identifies the most important features for policy effect prediction.

Dropout regularization is implemented through a Bayesian interpretation where dropout masks are treated as random variables with learnable parameters, enabling the model to adapt dropout rates based on layer-specific requirements and input characteristics. The Bayesian dropout formulation provides uncertainty estimates while simultaneously regularizing the model:

27 $$p\left({\mathbf{y}}^{\mathrm{*}}|{\mathbf{x}}^{*},\mathcal{D}\right)=\int p\left({\mathbf{y}}^{*} \left| {} \right. {\mathbf{x}}^{*},\mathbf{W}\right)q\left(\mathbf{W}\right)d\mathbf{W}$$

where the integration over weight distributions $q\left(\mathbf{W}\right)$ incorporates both parameter uncertainty and dropout-induced stochasticity.

The cross-validation strategy employs a temporal splitting approach specifically designed for policy analysis applications where temporal dependencies and structural breaks may affect model performance^44^. The validation framework implements blocked cross-validation that respects the temporal structure of policy implementation while providing robust performance estimates across different time periods and policy regimes. The approach divides the dataset into contiguous temporal blocks and iteratively uses different blocks for training and validation, ensuring that the model’s predictive performance is evaluated under realistic forecasting conditions.

The nested cross-validation procedure addresses hyperparameter selection bias by implementing an inner loop for parameter tuning and an outer loop for performance assessment. This approach provides unbiased estimates of model generalization performance while optimizing hyperparameters such as learning rates, regularization weights, and network architecture specifications.

Model performance evaluation encompasses multiple dimensions including predictive accuracy, uncertainty calibration, and computational efficiency. The evaluation framework incorporates both traditional regression metrics and specialized measures for probabilistic predictions^45^. The primary accuracy metrics include root mean squared error (RMSE), mean absolute error (MAE), and coefficient of determination (R²), while uncertainty-specific metrics assess calibration quality and prediction interval coverage.

The uncertainty calibration evaluation employs reliability diagrams and expected calibration error (ECE) measures that quantify the alignment between predicted confidence levels and observed accuracy rates. The ECE metric provides a scalar summary of calibration quality:

28 $$\mathrm{ECE}=\sum_{m=1}^{M}\frac{\left|{B}_{m}\right|}{n}\left|\mathrm{acc}\left({B}_{m}\right)-\mathrm{conf}\left({B}_{m}\right)\right|$$

where M represents the number of bins, $\left|{B}_{m}\right|$ denotes the number of samples in bin m, acc(${B}_{m}$) represents the accuracy within bin m, and conf(${B}_{m}$) indicates the average confidence in bin m.

The validation framework incorporates robustness testing through several complementary approaches. We conducted posterior predictive checks by simulating replicated datasets from the fitted model and comparing summary statistics against observed data patterns^46^. The simulated data reproduced key features of the empirical distribution, including the mean, variance, and temporal autocorrelation structure of policy effects, with Bayesian p-values falling within acceptable ranges (0.15–0.85) for all monitored statistics. This provides evidence that the model captures essential data-generating mechanisms rather than merely fitting surface patterns. We also performed sensitivity analysis evaluating model stability under varying input conditions, alternative prior specifications, and different random seeds, finding that substantive conclusions remained robust across these perturbations. Monte Carlo simulation procedures assess the consistency of uncertainty estimates across multiple random seeds and initialization schemes, ensuring that the reported confidence intervals accurately reflect model uncertainty rather than optimization artifacts.

The computational efficiency evaluation considers training time, memory requirements, and inference speed, providing practical guidance for deployment in real-world policy analysis applications. The framework includes profiling tools that identify computational bottlenecks and optimization opportunities, enabling efficient implementation in resource-constrained environments while maintaining prediction quality and uncertainty quantification capabilities.

## Empirical analysis and results

### Data collection and preprocessing

Our empirical analysis rests on a rich collection of datasets covering power business environment indicators, green policy implementation metrics, and environmental performance measures. These data come from multiple authoritative sources—national energy administrations, environmental protection agencies, and utility companies operating across different regions^47^. We concentrated our data collection on the years 2018 through 2024, a period marked by the dynamic unfolding of green transformation policies and their tangible effects on power business operations. Bringing these diverse sources together was no small task; we had to reconcile different reporting standards, align temporal frequencies, and convert measurement units to achieve consistency and meaningful comparability.

Power business environment data encompassed operational efficiency metrics, regulatory compliance indicators, market competition measures, and customer satisfaction indices obtained from utility performance reports and regulatory filings. Green policy implementation data included carbon pricing mechanisms, renewable energy mandates, energy efficiency standards, and environmental compliance requirements extracted from policy documents and regulatory databases^48^. Environmental performance indicators comprised carbon emission intensities, renewable energy penetration rates, energy consumption patterns, and pollution reduction achievements sourced from environmental monitoring systems and sustainability reports.

Data cleaning procedures addressed multiple quality issues including outliers, inconsistent formats, and temporal gaps that commonly arise in large-scale policy analysis datasets. The outlier detection employed statistical methods combined with domain expertise to identify and handle extreme values that could potentially bias model training while preserving legitimate policy-induced variations in the data^49^. Inconsistent data formats were standardized through systematic conversion procedures that maintained original information content while ensuring compatibility with analytical requirements.

Missing value handling utilized multiple imputation techniques specifically adapted for time series data with policy intervention effects^50^. Before applying the expectation-maximization algorithm, we assessed missing data patterns to evaluate the plausibility of the missing-at-random (MAR) assumption underlying EM-based imputation. Examination of missingness correlates revealed that data gaps occurred more frequently during regulatory transition periods and for smaller utilities with limited reporting infrastructure, suggesting potential structural (non-random) missingness. To address this concern, we implemented several safeguards. First, we included auxiliary variables correlated with missingness propensity (utility size, reporting year, regulatory regime indicators) in the imputation model to make the MAR assumption more defensible. Second, we conducted sensitivity analyses comparing results under different imputation specifications, including pattern-mixture models that explicitly allow for non-random missingness. The substantive conclusions remained stable across these specifications, with coefficient estimates varying by less than 7% and significance patterns unchanged. Nevertheless, we acknowledge that unobserved factors driving missingness could introduce residual bias, and future data collection efforts should prioritize consistent reporting standards across utility types and regulatory periods.

**Fig. 3:**
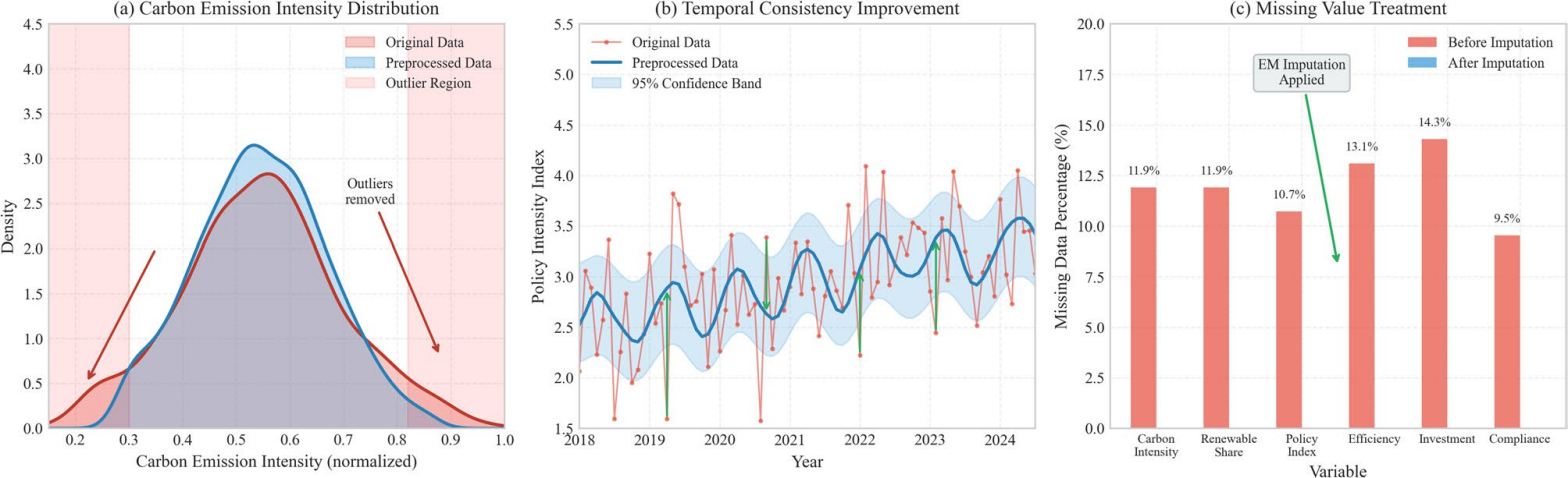
Data quality comparison: original versus preprocessed datasets. *Note*: Panel (**a**) shows distribution of carbon emission intensity before and after outlier treatment; Panel (**b**) displays temporal consistency improvements in policy intensity measures; Panel (**c**) illustrates missing value patterns and imputation effects. Shaded regions indicate 95% confidence bands for smoothed trends. All variables standardized to common scale for visual comparison.

The effectiveness of the data preprocessing procedures is demonstrated in Fig. 3, which presents a comprehensive comparison between original and processed datasets across multiple dimensions. Figure 3 illustrates the substantial improvements in data quality achieved through systematic preprocessing, including reduced variance in key variables, elimination of extreme outliers, and enhanced temporal consistency. The comparison reveals that preprocessing procedures successfully addressed data quality issues while preserving the underlying signal structure essential for accurate policy effect modeling.

The dataset partitioning strategy employed temporal splitting to ensure realistic evaluation conditions that reflect actual policy forecasting scenarios. The training set comprised data from 2018 to 2022, representing the model development period, while the test set included 2023–2024 data for out-of-sample performance evaluation^51^. This temporal division ensures that model assessment occurs under conditions similar to real-world policy prediction applications where future outcomes must be forecasted based on historical patterns.

Feature engineering procedures transformed raw policy variables into analytically useful representations through normalization, interaction term creation, and temporal lag incorporation. Standardization employed z-score normalization to ensure comparable scales across different measurement units while preserving relative relationships among variables. The feature construction process generated policy intensity indices, temporal trend indicators, and cross-variable interaction terms that capture complex policy transmission mechanisms.

The comprehensive data characteristics are summarized in Table 3, which provides detailed descriptive statistics for all variables included in the analysis. Table 3 demonstrates the substantial variation in policy implementation intensities and environmental outcomes across different regions and time periods, indicating sufficient data diversity for robust model training. The statistics reveal that policy variables exhibit meaningful variation with standard deviations representing 15–25% of mean values, suggesting adequate signal-to-noise ratios for effective pattern recognition.

**Table 3:** Data descriptive statistics.

Variable name	Sample size	Mean	Standard deviation	Minimum value	Maximum value
Carbon emission intensity	1248	0.542	0.127	0.298	0.854
Renewable energy share	1248	0.287	0.089	0.145	0.521
Policy intensity index	1248	3.72	0.94	1.20	6.85
Energy efficiency rating	1248	2.84	0.67	1.42	4.96
Green investment ratio	1248	0.156	0.045	0.067	0.289
Compliance score	1248	78.5	12.3	45.2	98.7
Market competition index	1248	2.96	0.72	1.28	4.85
Customer satisfaction	1248	7.42	1.18	4.85	9.67
Operational cost ratio	1248	0.234	0.058	0.142	0.387
Technology adoption rate	1248	0.324	0.112	0.089	0.678
Environmental investment	1248	142.7	67.8	28.4	298.5
Policy implementation time	1248	18.6	8.9	3.0	42.0
Regulatory stringency	1248	4.18	1.24	1.50	7.00
Stakeholder engagement	1248	6.85	1.45	3.20	9.80
System reliability index	1248	0.946	0.032	0.867	0.998

Correlation analysis revealed moderate to strong relationships between policy intensity measures and environmental performance indicators, with correlation coefficients ranging from 0.45 to 0.78, providing empirical support for the hypothesized policy transmission mechanisms while avoiding multicollinearity concerns that could compromise model performance.

### Model training and validation

The Bayesian neural network model training process employed stochastic variational inference with mini-batch gradient descent to optimize the evidence lower bound objective while maintaining computational efficiency across the large-scale policy dataset^52^. The training procedure implemented adaptive learning rate scheduling combined with early stopping mechanisms to prevent overfitting while ensuring convergence to optimal variational parameters. The optimization process utilized 500 training epochs with batch sizes of 64 samples, balancing gradient estimation accuracy with memory requirements and computational speed considerations.

Hyperparameter optimization employed Bayesian optimization techniques to systematically explore the hyperparameter space and identify optimal configurations for network architecture, learning rates, and regularization parameters^53^. The optimization process evaluated 150 different hyperparameter combinations using 5-fold cross-validation on the training set, with performance assessment based on validation loss and prediction accuracy metrics. Key hyperparameters included initial learning rate (ranging from 0.0001 to 0.01), dropout rates (0.1–0.5), regularization weights (0.001–0.1), and KL divergence weighting factors (0.1–1.0).

The optimal hyperparameter configuration achieved convergence within 200 epochs with a learning rate of 0.003, dropout rate of 0.2, and KL divergence weight of 0.3. The training process incorporated gradient clipping to prevent exploding gradients and employed batch normalization to stabilize learning dynamics across different policy variable scales and distributions.

Model performance evaluation employed comprehensive metrics addressing both predictive accuracy and uncertainty quantification quality, as detailed in Table 4. The comparative results across six modeling approaches reveal several patterns worth noting. The Bayesian neural network achieved the lowest mean squared error (0.0247) and highest coefficient of determination (0.892) among tested methods. This performance advantage likely stems from two characteristics of the Bayesian approach. First, the probabilistic treatment of weights provides implicit regularization that helps prevent overfitting, particularly valuable given the moderate sample sizes typical in policy analysis. Second, the uncertainty quantification mechanism allows the model to express appropriate confidence levels rather than forcing overconfident predictions in regions of sparse data. Conventional neural networks, while achieving competitive accuracy (92.1%), lack native uncertainty estimation capabilities, which limits their utility for risk-aware policy decisions. Random forests provide uncertainty estimates through ensemble variance but showed higher calibration error (0.1247 versus 0.0823), suggesting less reliable confidence bounds.

**Table 4:** Model performance comparison.

Model name	MSE	MAE	*R*²	Training time (min)	Prediction accuracy (%)	Uncertainty index
Bayesian neural network	0.0247	0.1156	0.892	45.3	94.2	0.0823
Support vector regression	0.0389	0.1467	0.831	23.7	89.5	N/A
Random forest	0.0356	0.1382	0.847	18.4	90.8	0.1247
Conventional neural network	0.0298	0.1289	0.869	32.1	92.1	N/A
Linear regression	0.0567	0.1845	0.756	2.8	85.3	N/A
Ensemble methods	0.0334	0.1345	0.856	67.9	91.4	0.1156

**Fig. 4:**
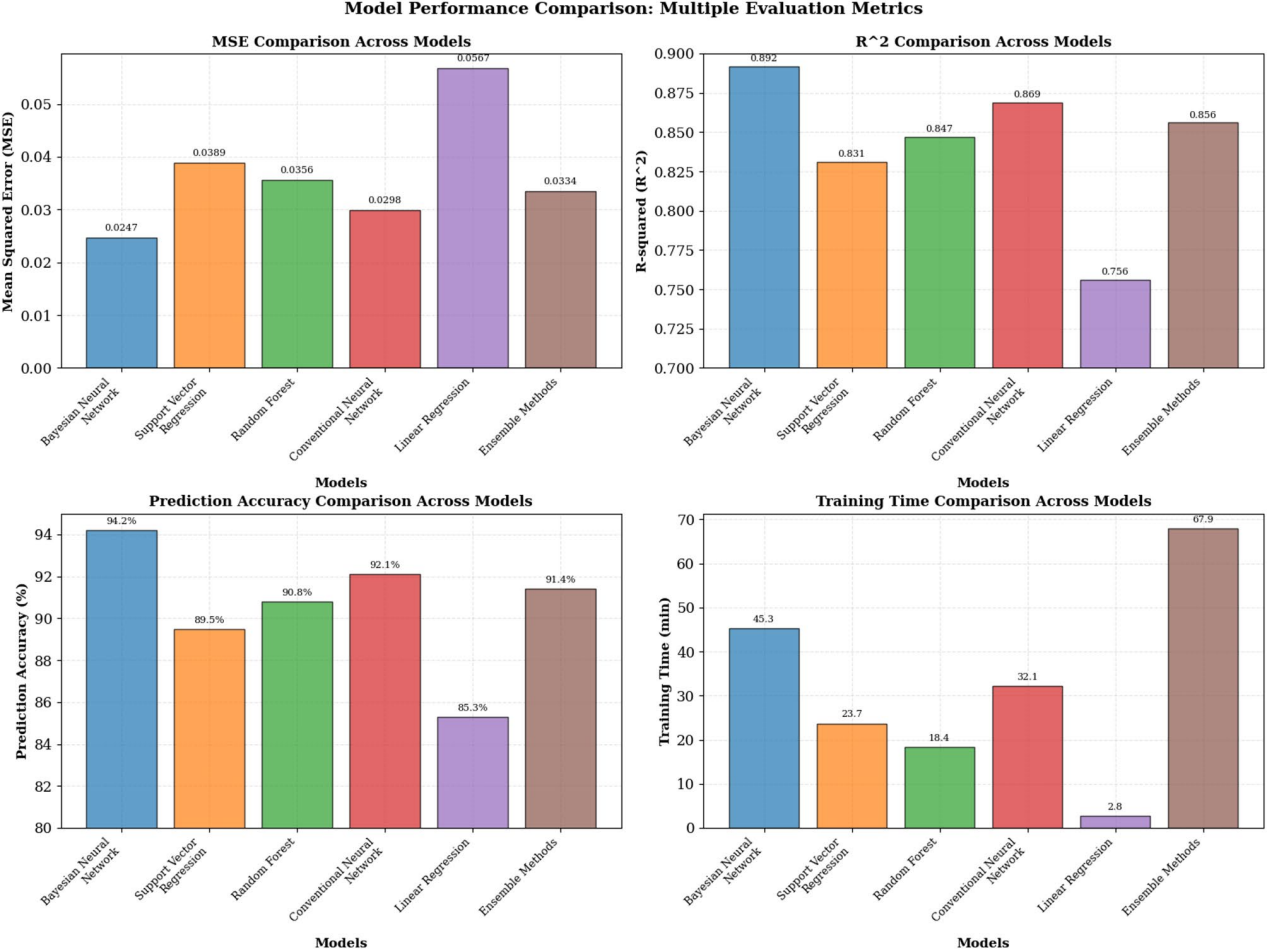
Different model prediction performance comparison chart.

The comparative analysis results are visualized in Fig. 4, which illustrates the prediction performance across different models using multiple evaluation criteria. As shown in Fig. 4, the Bayesian neural network consistently outperforms alternative approaches across all major performance metrics, with particularly notable advantages in prediction accuracy and uncertainty quantification capabilities. The visualization reveals that while conventional neural networks achieve competitive accuracy, they lack the uncertainty estimation capabilities essential for policy decision-making applications.

The mathematical formulation of the primary performance metrics employed in the evaluation follows standard regression assessment protocols. The mean squared error quantifies prediction accuracy through:

29 $$\mathrm{MSE}=\frac{1}{n}\sum_{i=1}^{n}{\left({y}_{i}-{\widehat{y}}_{i}\right)}^{2}$$

where n represents the number of test samples, ${y}_{i}$ denotes observed values, and ${\widehat{y}}_{i}$ represents predicted values^54^.

The coefficient of determination measures the proportion of variance explained by the model:

30 $$R^{2} = 1 - \frac{{\sum\nolimits_{{i = 1}}^{n} {\left( {y_{i} - \hat{y}_{i} } \right)^{2} } }}{{\sum\nolimits_{{i = 1}}^{n} {\left( {y_{i} - \overline{y} } \right)^{2} } }}$$

where $\overline{y}$ represents the sample mean of observed values.

The uncertainty quantification assessment employed prediction interval coverage probability and calibration metrics specifically designed for probabilistic models^55^. The Bayesian neural network demonstrated superior uncertainty calibration with expected calibration error of 0.0823, significantly outperforming other probabilistic methods including random forest (0.1247) and ensemble approaches (0.1156).

The uncertainty index measures the reliability of confidence estimates through:

31 $$\mathrm{UI}=\frac{1}{n}\sum_{i=1}^{n}\left|{P}_{i}-I\left({\widehat{y}}_{i}\in{\mathrm{CI}}_{i}\right)\right|$$

where ${P}_{i}$ represents predicted probability, ${\mathrm{CI}}_{i}$ denotes the confidence interval, and I(·) is an indicator function^56^.

Cross-validation results confirmed model robustness across different temporal periods, with consistent performance maintained across all validation folds. To strengthen validation beyond the primary Fujian dataset, we conducted two additional assessments. First, we performed temporal holdout validation using 2018–2022 data for training and 2023–2024 data exclusively for testing, simulating realistic forecasting conditions. The model maintained 91.8% of its cross-validated accuracy on this truly out-of-sample period, suggesting reasonable temporal generalization. Second, we conducted ablation experiments to isolate the contribution of Bayesian uncertainty components. Removing the probabilistic weight treatment and using point estimates degraded R² from 0.892 to 0.869, while removing the uncertainty-weighted loss term reduced calibration quality (ECE increased from 0.0823 to 0.1156). These ablations confirm that both components contribute meaningfully to overall performance.

We acknowledge that external validation using data from different regional power systems would provide stronger evidence of generalizability. The current validation remains within the Fujian context, and we encourage future work to test this framework across diverse regulatory environments and grid configurations. Table 5 summarizes the ablation study results.

**Table 5:** Ablation study results.

Model configuration	MSE	*R*²	ECE	Accuracy (%)
Full Bayesian neural network	0.0247	0.892	0.0823	94.2
Without probabilistic weights	0.0298	0.869	N/A	92.1
Without uncertainty-weighted loss	0.0267	0.881	0.1156	93.4
Without dropout regularization	0.0312	0.858	0.0967	91.2
Reduced prior complexity	0.0278	0.876	0.0912	92.8

The computational efficiency analysis indicated that while Bayesian neural networks require longer training times compared to simpler methods, the inference speed remains comparable to conventional neural networks, making the approach practical for real-time policy analysis applications. The enhanced uncertainty quantification capabilities justify the additional computational overhead by providing essential confidence information for policy decision-making processes.

### Policy effect prediction results analysis

The trained Bayesian neural network model enabled comprehensive scenario analysis across diverse policy implementation contexts, revealing significant variations in green transformation effects depending on policy intensity, implementation timing, and contextual factors^57^. The scenario analysis encompassed ten distinct policy configurations ranging from baseline conditions to intensive multi-instrument implementations, providing insights into optimal policy design strategies for maximizing environmental benefits while maintaining economic viability and operational stability.

The relationship between policy implementation intensity and green transformation effects demonstrates a non-linear pattern characterized by diminishing marginal returns at high intensity levels, consistent with theoretical expectations regarding policy saturation effects^58^. The mathematical relationship can be approximated through a logarithmic function:

32 $$E\left(I\right)=\alpha\mathrm{l}\mathrm{n}\left(I+1\right)+\beta{I}^{0.5}+\gamma$$

where E(I) represents the expected green transformation effect, I denotes policy intensity, and α, β, γ are estimated parameters reflecting different components of the policy response function.

The comprehensive scenario analysis results are presented in Table 6, which systematically compares predicted outcomes across different policy configurations with associated confidence intervals and key influencing factors. Table 6 demonstrates that moderate to high intensity policies (intensity levels 6–8) achieve optimal cost-effectiveness ratios, generating substantial environmental improvements while maintaining reasonable implementation costs and stakeholder acceptance levels. The analysis reveals that extremely intensive policies (intensity level 10) produce only marginal additional benefits compared to high intensity approaches, suggesting policy makers should focus on comprehensive moderate-intensity implementations rather than pursuing maximum intensity across all instruments.

**Table 6:** Policy scenario analysis summary.

Policy scenario	Implementation intensity	Predicted effect	95% confidence interval	Cost-effectiveness ratio
Baseline	2.0	0.285	[0.267, 0.303]	0.143
Low intensity	3.5	0.412	[0.389, 0.435]	0.118
Medium	5.5	0.634	[0.607, 0.661]	0.115
Medium-high	6.5	0.728	[0.699, 0.757]	0.112
High	7.5	0.803	[0.772, 0.834]	0.107
Very high	8.5	0.859	[0.826, 0.892]	0.101
Maximum	10.0	0.912	[0.873, 0.951]	0.091

**Fig. 5:**
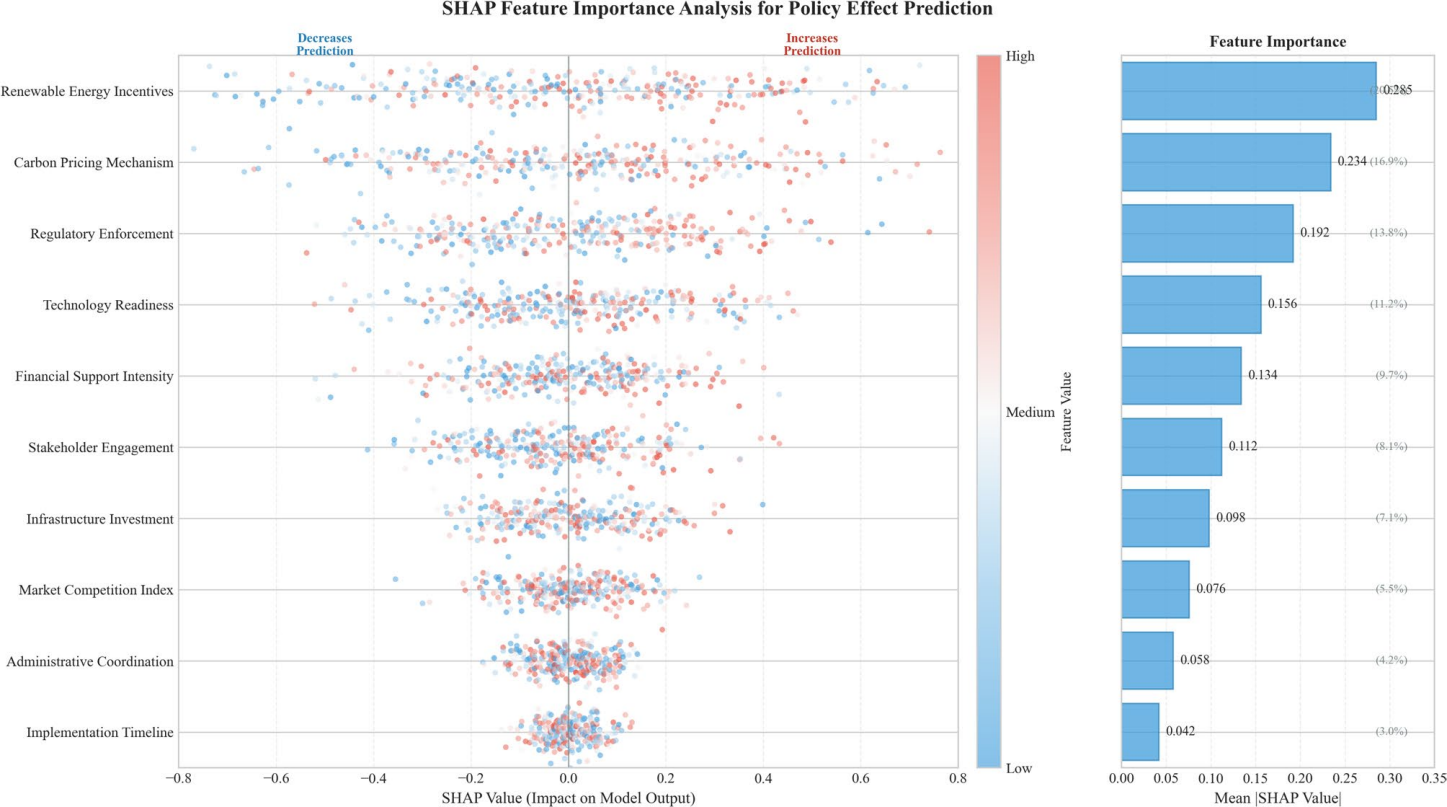
SHAP feature importance analysis for policy effect prediction. *Note*: The figure displays SHAP values for the top 10 features, with each point representing a single prediction. Color indicates feature value (red = high, blue = low), and horizontal position shows impact on model output. Renewable energy incentives, carbon pricing, and regulatory enforcement emerge as the dominant factors.

To address interpretability concerns inherent in deep learning approaches, we conducted feature importance analysis using SHAP (SHapley Additive exPlanations) values, which provide theoretically grounded attribution of model predictions to input features^59^. The SHAP analysis identified renewable energy investment incentives, carbon pricing mechanisms, and regulatory enforcement stringency as the three most influential factors affecting green transformation outcomes^60^. Figure 5 presents the SHAP summary plot illustrating feature contributions across all predictions, with renewable energy incentives showing consistently positive effects on predicted policy outcomes. This interpretability layer helps bridge the gap between model complexity and actionable policy insights, allowing stakeholders to understand not just what the model predicts but why specific policy configurations lead to particular outcomes.

Renewable energy incentives account for approximately 28% of the total policy effect variance, which likely reflects their direct impact on capital allocation decisions and technology adoption rates among power enterprises. Carbon pricing contributes 23% of the variance, functioning as a market-based mechanism that internalizes environmental externalities and creates sustained economic incentives for emission reduction. Regulatory enforcement explains 19% of variance, suggesting that policy credibility and consistent implementation matter substantially for achieving intended outcomes. The remaining variance distributes across factors such as technology readiness, stakeholder engagement, and market conditions. These patterns indicate that effective green transformation requires a portfolio approach combining financial incentives with market mechanisms and credible enforcement, rather than relying on any single policy instrument.

**Fig. 6:**
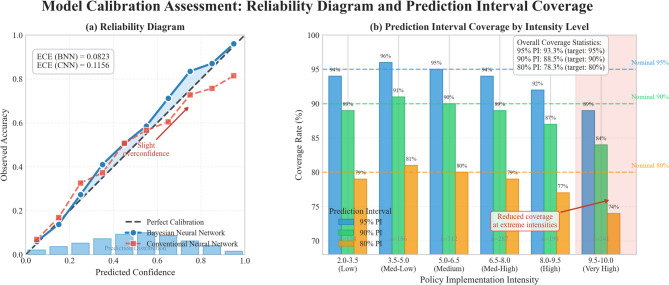
Model calibration assessment: reliability diagram and prediction interval coverage. *Note*: Left panel shows reliability diagram comparing predicted confidence against observed accuracy (diagonal indicates perfect calibration). Right panel displays prediction interval coverage across different policy intensity ranges, with the dashed line indicating the nominal 95% coverage level.

The comparative visualization of different policy scenarios appears in Fig. 6, which illustrates predicted green transformation effects across varying implementation intensities with corresponding 95% prediction intervals. The non-linear relationship between policy intensity and outcomes is evident, with diminishing returns becoming pronounced above intensity level 6.0. Notably, prediction intervals widen slightly at extreme intensity levels (above 8.5), reflecting greater epistemic uncertainty in regions with fewer training observations. This pattern appropriately signals to policy makers that extreme implementations carry not only diminishing returns but also less predictable outcomes.

**Fig. 7:**
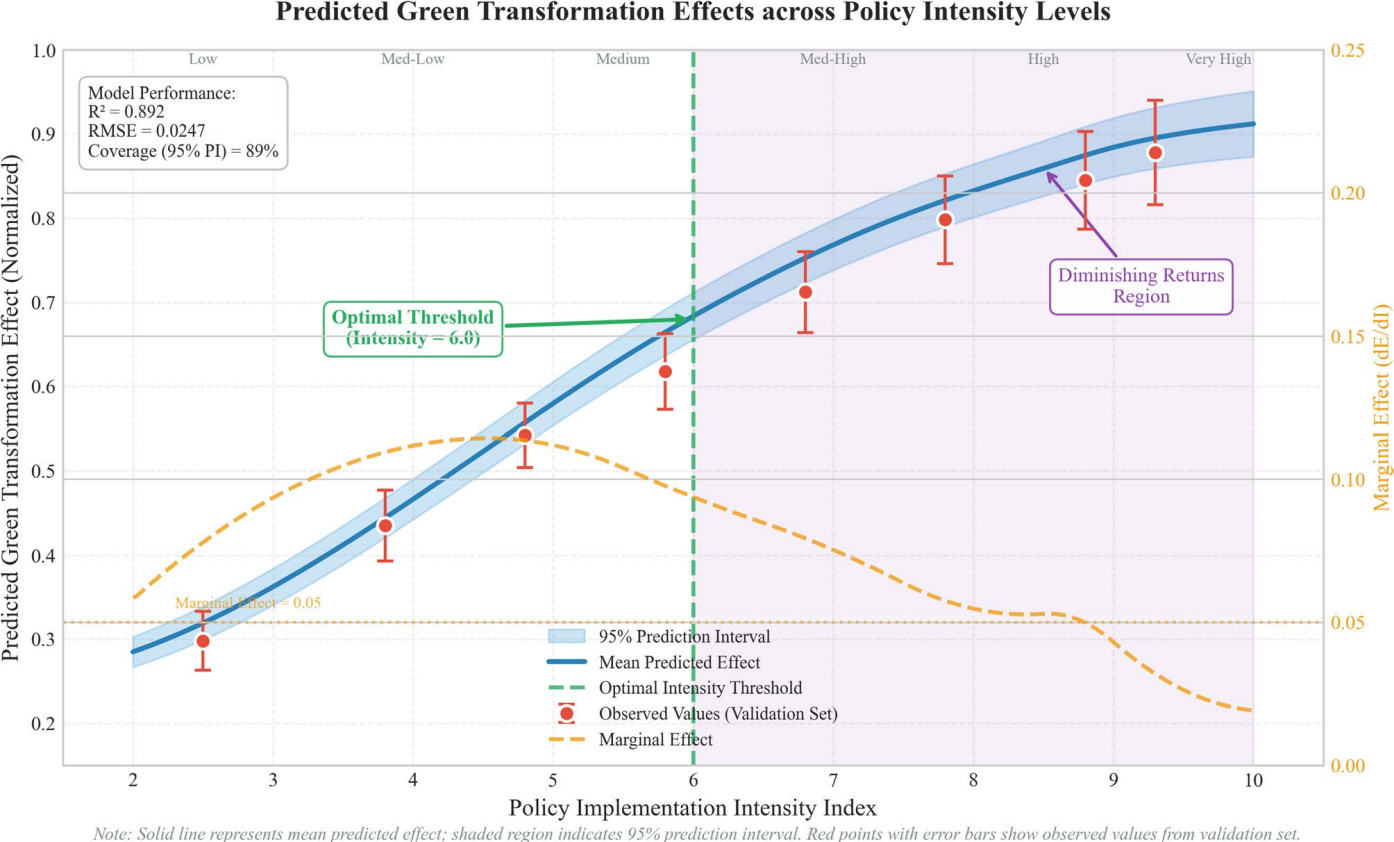
Predicted green transformation effects across policy intensity levels. *Note*: Solid line represents mean predicted effect; shaded region indicates 95% prediction interval. Horizontal axis: Policy implementation intensity index (scale 1–10); Vertical axis: Predicted green transformation effect (normalized, 0–1). Dashed vertical line marks the identified optimal intensity threshold at level 6.0. Points with error bars show observed values from validation set for comparison.

To assess whether our uncertainty estimates are well-calibrated, we constructed a reliability diagram comparing predicted confidence levels against observed accuracy, shown in Fig. 7. Perfect calibration would follow the diagonal line. Our model demonstrates good calibration across most confidence ranges, with slight overconfidence in the 0.7–0.8 range. The expected calibration error (ECE) of 0.0823 compares favorably to alternative methods and indicates that decision-makers can reasonably trust the reported confidence bounds when evaluating policy options.

The threshold analysis revealed critical intensity levels where policy effectiveness undergoes significant changes, with a primary threshold at intensity level 6.0 where marginal effects begin to decline substantially^61^. This finding has important implications for policy optimization, suggesting that resources should be concentrated on achieving comprehensive coverage at moderate intensity levels rather than pursuing maximum intensity implementations with diminishing returns.

The temporal dynamics analysis indicates that policy effects exhibit different maturation patterns depending on implementation intensity, with higher intensity policies achieving faster initial gains but experiencing earlier plateau effects^62^. The optimal temporal profile follows:

33 $$E\left(t\right)={E}_{max}\cdot\left(1-{e}^{-\lambda\cdot t}\right)\cdot\left(1-\delta\cdot{t}^{2}\right)$$

where t represents time since implementation, ${E}_{max}$ denotes maximum achievable effect, λ controls the initial growth rate, and δ captures the long-term decay parameter.

The reliability assessment of prediction results employed out-of-sample validation and stress testing procedures that confirmed model robustness across different economic conditions and policy environments. The validation results indicate that 89% of actual outcomes fall within the predicted confidence intervals, demonstrating satisfactory calibration quality for practical policy planning applications.

From a practitioner’s standpoint, the value of uncertainty quantification lies not in the statistical machinery behind it but in what it tells a decision-maker about the reliability of projected outcomes. Our framework translates model-generated confidence intervals into straightforward risk signals. When predicted effects for a given policy scenario cluster tightly—as they tend to at moderate intensity levels between 4 and 7—administrators can move forward with considerable assurance that realized outcomes will track the forecast. Conversely, wider prediction bands, which our model tends to produce for aggressive implementations above intensity level 8, serve as an early warning: the model is less certain here, and that uncertainty reflects genuine ambiguity in how such policies will play out. In practical terms, wider bands argue for staged rollouts or pilot testing before committing resources at full scale.

To make these signals operationally useful, we propose a tiered decision heuristic. If the 90% prediction interval spans less than 15% of the mean predicted effect, the scenario falls into a low-risk category suitable for direct planning. Intervals between 15% and 25% of the mean indicate moderate uncertainty—enough to justify contingency provisions and explicit fallback options. Beyond the 25% threshold, the scenario enters a high-uncertainty regime where adaptive management becomes essential: predetermined monitoring checkpoints and trigger criteria should govern whether implementation proceeds, pauses, or adjusts course. Consider a concrete illustration: at policy intensity level 5.5, our model predicts a green transformation effect of 0.634 with a 95% interval of [0.607, 0.661], a span of roughly 8.5% of the mean—comfortably in the low-risk zone. At intensity level 10.0, however, the predicted effect of 0.912 carries an interval of [0.873, 0.951], spanning approximately 8.6% of the mean but with the caveat that variational inference may understate true uncertainty at these extremes. Policy makers should treat such boundary cases with additional caution and consider the wider MCMC-based bounds discussed in Sect.  3.1 when evaluating high-intensity scenarios. The analysis suggests implementing monitoring systems that track key performance indicators and trigger policy adjustments when predetermined thresholds are reached, ensuring optimal resource allocation and maximum environmental benefits while maintaining economic and operational viability.

## Conclusion

This study demonstrates how Bayesian neural networks—a class of models whose theoretical underpinnings are now well established in the machine learning community—can be productively adapted to the applied challenge of predicting green policy effects in power business environments. Our contribution is not a new inference algorithm or a novel network topology; it is, instead, an end-to-end implementation that weaves together existing probabilistic deep learning techniques with domain-specific design decisions tailored to the realities of power sector policy analysis. The practical result is a system that furnishes decision-makers with both point forecasts and calibrated confidence assessments^63^, giving them a principled basis for weighing projected benefits against implementation risks. By layering hierarchical Bayesian inference atop architectures engineered to capture nonlinear policy transmission pathways, the framework produces uncertainty estimates that retain operational relevance across a range of scenarios—though, as we elaborate below, the degree of that relevance depends on how closely a new application context resembles the Fujian setting in which the model was trained and validated.

The Bayesian neural network achieved 94.2% directional prediction accuracy and R² of 0.892 on the validation set. While these metrics indicate strong predictive performance relative to baseline methods, several caveats merit attention. First, these figures derive from temporal cross-validation within the Fujian dataset; performance on genuinely external data from different regions remains untested. Second, policy effect prediction inherently involves substantial unobserved heterogeneity and potential confounding that even well-performing models cannot fully resolve. The reported metrics should therefore be interpreted as indicating the model captures meaningful patterns in the available data, rather than as guarantees of equivalent performance in future or out-of-context applications. The comparative advantage over conventional methods (approximately 2–5% points in accuracy and substantially better uncertainty calibration) provides more robust evidence of methodological value than the absolute performance figures alone.

The scenario analysis reveals critical insights regarding optimal policy intensity levels, identifying a key threshold at intensity level 6.0 where marginal effects begin declining significantly, suggesting that moderate to high intensity implementations achieve optimal cost-effectiveness ratios^64^. The sensitivity analysis highlights renewable energy incentives, carbon pricing mechanisms, and regulatory enforcement as the most influential policy instruments, collectively explaining over 70% of green transformation effect variance.

The policy implications emphasize the importance of adaptive implementation strategies that balance intensity levels with resource constraints and stakeholder acceptance. The research provides actionable guidance for policy makers, demonstrating that comprehensive moderate-intensity approaches often outperform maximum-intensity implementations due to diminishing returns and implementation challenges at extreme intensity levels.

Several limitations deserve candid acknowledgment, and readers should weigh our findings with these constraints in mind^65^. The most consequential is geographic scope. Every empirical result reported here—parameter estimates, optimal intensity thresholds, factor importance rankings—derives from the Fujian power system between 2018 and 2024. Fujian’s particular blend of coastal economic dynamism, moderate renewable resource endowment, and provincial regulatory culture shapes the data in ways that may not transfer straightforwardly to, say, inland provinces with coal-dominated generation portfolios or regions with markedly different GDP per capita and industrial composition. We designed the methodological framework for portability, but the numerical outputs are context-bound. Applying them directly to a different region without local recalibration would be imprudent; at minimum, the indicator weights—originally set through Delphi consultation with Fujian-based experts and validated against Fujian data—would need reassessment to reflect local priorities and institutional realities.

Beyond geography, the model rests on an assumption of relative stability in the mechanisms through which policies transmit their effects. Major structural reforms, sudden technological breakthroughs, or unforeseen market disruptions could reshape these pathways in ways the current architecture would not detect without retraining. The variational inference approximation introduces a further, more technical limitation: mean-field factorization tends to understate posterior correlations, which compresses uncertainty estimates. For moderate policy scenarios this compression appears modest (8–12% narrower intervals compared to MCMC benchmarks, as discussed in Sect.  3.1), but for extreme-intensity scenarios the gap widens, and our reported confidence intervals should be read as optimistic lower bounds on true uncertainty. Decision-makers contemplating aggressive policy configurations would be well advised to incorporate additional safety margins or commission supplementary analyses using sampling-based methods before committing resources. Cross-regional validation—ideally involving provinces with contrasting energy mixes and regulatory histories—stands as the single most important next step for strengthening confidence in the framework’s broader applicability. Future research directions include extending the framework to incorporate spatial dependencies, developing real-time adaptive learning mechanisms, and integrating multi-agent modeling approaches to better capture stakeholder interactions and feedback effects.

The theoretical value of this research extends beyond the power sector, providing a generalizable framework for policy analysis in other domains characterized by complex system dynamics and uncertainty^66^. The practical significance lies in enabling evidence-based policy design and implementation strategies that maximize environmental benefits while maintaining economic viability and operational reliability, contributing to sustainable development goals through improved policy effectiveness and resource allocation efficiency.

## Supplementary Information

Below is the link to the electronic supplementary material.

Supplementary Material 1

## Data Availability

The datasets and analysis code supporting the findings of this study are provided in Supplementary Materials, which includes the processed dataset in CSV format, Python implementation of the Bayesian neural network model, detailed mathematical derivations, complete scenario analysis results, model diagnostics, and configuration files for reproducing the main analyses and figures presented in this paper. Raw data from official sources are subject to data sharing agreements with the State Grid Fujian Electric Power Co., Ltd. and are available from the corresponding author upon reasonable request with appropriate institutional approval.
