# Supplementary material for: Bayesian neural network-based policy effect prediction for green transformation of power business environment
Source: Sci Rep. 2026 Mar 7;16:12502. doi: 10.1038/s41598-026-42092-z (PMC13087194; doi:10.1038/s41598-026-42092-z)
Supplement: Supplementary file 1 — Supplementary Material 1 [file 41598_2026_42092_MOESM1_ESM.docx]

# Supplementary Materials

# Supplementary Appendix A: Fundamental Mathematical Derivations

This appendix presents the foundational equations referenced in the main text as Equations (1)-(3), along with supporting derivations for Bayesian inference in neural networks.

## A.1 Likelihood Function Derivation (Equation 1)

For a dataset D = {(x₁, y₁), (x₂, y₂), …, (xₙ, yₙ)}, assuming Gaussian noise with variance σ², the likelihood function for policy effect observations is:

$$P\left( D|\theta\right)=\prod_{i=1}^{N} P\left( y_{i}|x_{i},\theta\right)=\prod_{i=1}^{N} \mathcal{N}\left( y_{i}|f_{\theta}\left( x_{i} \right),\sigma^{2} \right) \text{(Equation 1)}$$

The log-likelihood can be expressed as:

$$\log P\left( D|\theta\right)=-\frac{N}{2}\log\left( 2\pi\sigma^{2} \right)-\frac{1}{2\sigma^{2}}\sum_{i=1}^{N} \left( y_{i}-f_{\theta}\left( x_{i} \right) \right)^{2}$$

For heteroscedastic models where noise variance varies with input:

$$P\left( y_{i}|x_{i},\theta\right)\mathcal{=N}\left( y_{i}|\mu_{\theta}\left( x_{i} \right),\sigma_{\theta}^{2}\left( x_{i} \right) \right)$$

The negative log-likelihood becomes:

$$-logP\left( D|\theta\right)=\frac{1}{2}\sum_{i=1}^{N} \left[ \log\sigma_{\theta}^{2}\left( x_{i} \right)+\frac{\left( y_{i}-\mu_{\theta}\left( x_{i} \right) \right)^{2}}{\sigma_{\theta}^{2}\left( x_{i} \right)} \right]+\frac{N}{2}\log\left( 2\pi\right)$$

## A.2 Posterior Distribution Computation (Equation 2)

Using Bayes’ theorem, the posterior distribution over network parameters is:

$$P\left( \theta|D \right)=\frac{P\left( D|\theta\right)P\left( \theta\right)}{P\left( D \right)}=\frac{P\left( D|\theta\right)P\left( \theta\right)}{\int P\left( D|\theta\right)P\left( \theta\right)d\theta} \text{(Equation 2)}$$

The marginal likelihood (evidence) P(D) serves as a normalizing constant:

$$P\left( D \right)=\int P\left( D|\theta\right)P\left( \theta\right)d\theta$$

For neural networks, this integral is intractable. We approximate using variational inference by introducing a tractable distribution q(θ) and minimizing the KL divergence:

$$\text{KL}\left[ q\left( \theta\right)\left| \right|P\left( \theta|D \right) \right]=\int q\left( \theta\right)\log\frac{q\left( \theta\right)}{P\left( \theta|D \right)}d\theta$$

This leads to maximizing the Evidence Lower Bound (ELBO):

$$\mathcal{L}_{ELBO}=\mathbb{E}_{q\left( \theta\right)}\left[ \log P\left( D|\theta\right) \right]-\text{KL}\left[ q\left( \theta\right)\left| \right|P\left( \theta\right) \right]$$

## A.3 Predictive Distribution Derivation (Equation 3)

The predictive distribution for a new input x* is obtained by marginalizing over the posterior:

$$P\left( y^{*}|x^{*},D \right)=\int P\left( y^{*}|x^{*},\theta\right)P\left( \theta|D \right)d\theta\text{(Equation 3)}$$

Under the variational approximation:

$$P\left( y^{*}|x^{*},D \right)\approx\int P\left( y^{*}|x^{*},\theta\right)q\left( \theta\right)d\theta$$

Using Monte Carlo sampling with T samples:

$$P\left( y^{*}|x^{*},D \right)\approx\frac{1}{T}\sum_{t=1}^{T} P\left( y^{*}|x^{*},\theta_{t} \right), \theta_{t}\sim q\left( \theta\right)$$

The predictive mean and variance are:

$$\mathbb{E}\left[ y^{*} \right]\approx\frac{1}{T}\sum_{t=1}^{T} f_{\theta_{t}}\left( x^{*} \right)$$

$$\text{Var}\left[ y^{*} \right]\approx\frac{1}{T}\sum_{t=1}^{T} f_{\theta_{t}}\left( x^{*} \right)^{2}-\left( \frac{1}{T}\sum_{t=1}^{T} f_{\theta_{t}}\left( x^{*} \right) \right)^{2}+\frac{1}{T}\sum_{t=1}^{T} \sigma_{\theta_{t}}^{2}\left( x^{*} \right)$$

## A.4 KL Divergence for Gaussian Distributions

For weight matrices with Gaussian priors and posteriors:

Prior: $P\left( W \right)=\mathcal{N}\left( 0,\alpha^{-1}I \right)$

Posterior: $q\left( W \right)=\mathcal{N}\left( \mu_{W},\text{diag}\left( \sigma_{W}^{2} \right) \right)$

The KL divergence is:

$$\text{KL}\left[ q\left( W \right)\left| \right|P\left( W \right) \right]=\frac{1}{2}\sum_{j} \left[ \alpha\sigma_{W,j}^{2}+\alpha\mu_{W,j}^{2}-1-log\left( \alpha\sigma_{W,j}^{2} \right) \right]$$

# Supplementary Appendix B: Variational Parameter Gradient Derivations

This appendix provides the detailed gradient derivations for variational parameters referenced in Section III.1 of the main text.

## B.1 Reparameterization Trick

To enable gradient-based optimization through stochastic nodes, we reparameterize the random variable:

$$W=\mu_{W}+\sigma_{W}\odot\epsilon, \epsilon\mathcal{\sim N}\left( 0,I \right)$$

where $\odot$ denotes element-wise multiplication.

## B.2 Variational Posterior Parameterization

The variational posterior for weights takes the form:

$$q\left( W_{l} \right)\mathcal{=N}\left( \mu_{W_{l}},\text{diag}\left( \sigma_{W_{l}}^{2} \right) \right)$$

$$q\left( b_{l} \right)\mathcal{=N}\left( \mu_{b_{l}},\text{diag}\left( \sigma_{b_{l}}^{2} \right) \right)$$

where $\mu_{W_{l}}$, $\sigma_{W_{l}}$, $\mu_{b_{l}}$, and $\sigma_{b_{l}}$ represent the variational parameters to be optimized during training.

## B.3 Gradient Computations for Mean Parameters

The gradient with respect to the variational mean parameters:

$$\nabla_{\mu_{W}}\mathcal{L=}\nabla_{W}\mathcal{L}|_{W=\mu_{W}+\sigma_{W}\odot\epsilon}$$

The full gradient expression for mean parameters follows:

$$\nabla_{\mu}\mathcal{L}_{ELBO}=\mathbb{E}_{\epsilon}\left[ \nabla_{\theta}\log P\left( D|\theta\right)+\nabla_{\theta}\log P\left( \theta\right)-\nabla_{\theta}\log q\left( \theta\right) \right]$$

## B.4 Gradient Computations for Variance Parameters

The gradient with respect to the variational standard deviation parameters:

$$\nabla_{\sigma_{W}}\mathcal{L=}\epsilon\odot\nabla_{W}\mathcal{L}|_{W=\mu_{W}+\sigma_{W}\odot\epsilon}$$

The full gradient expression for variance parameters follows:

$$\nabla_{\sigma}\mathcal{L}_{ELBO}=\mathbb{E}_{\epsilon}\left[ \epsilon\cdot\left( \nabla_{\theta}\log P\left( D|\theta\right)+\nabla_{\theta}\log P\left( \theta\right)-\nabla_{\theta}\log q\left( \theta\right) \right) \right]$$

## B.5 Practical Implementation Notes

In practice, the expectations are estimated using a single Monte Carlo sample per mini-batch during training, which provides an unbiased gradient estimate with manageable variance. The log-variance parameterization $\rho=log\left( \sigma^{2} \right)$ is often preferred for numerical stability:

$$\sigma=\sqrt{\exp\left( \rho\right)}=exp\left( \rho/2 \right)$$

This ensures positivity of variance while allowing unconstrained optimization of $\rho$.

# Supplementary Appendix C: Policy Effect Evaluation Model Specification

This appendix provides the complete mathematical specification for the policy effect evaluation model referenced in Section III.2 of the main text.

## C.1 Primary Model Equation

The core mathematical model for policy effect evaluation employs a multi-level hierarchical structure. The primary model equation takes the form:

$$PE\left( t \right)=f\left( \mathbf{P}\left( t \right),\mathbf{E}\left( t \right),\mathbf{M}\left( t \right),\mathbf{C}\left( t \right) \right)+\varepsilon\left( t \right)$$

where:

- PE(t) represents the aggregated policy effect at time t
- **P**(t) denotes the policy implementation intensity vector
- **E**(t) represents environmental context variables
- **M**(t) captures market condition factors
- **C**(t) includes control variables
- ε(t) represents stochastic error terms

## C.2 Policy Implementation Intensity Vector

The policy implementation intensity vector **P**(t) comprises multiple policy dimensions:

$$\mathbf{P}\left( t \right)=\left[ P_{RE}\left( t \right),P_{CP}\left( t \right),P_{EE}\left( t \right),P_{RG}\left( t \right),P_{RD}\left( t \right),P_{GP}\left( t \right),P_{ID}\left( t \right),P_{SE}\left( t \right) \right]^{T}$$

where:

- $P_{RE}(t)$: Renewable energy subsidy intensity
- $P_{CP}(t)$: Carbon pricing mechanism strength
- $P_{EE}(t)$: Energy efficiency standard stringency
- $P_{RG}(t)$: Regulatory enforcement level
- $P_{RD}(t)$: R&D incentive magnitude
- $P_{GP}(t)$: Green procurement policy intensity
- $P_{ID}(t)$: Information disclosure requirements
- $P_{SE}(t)$: Stakeholder engagement program intensity

## C.3 Environmental Context Variables

$$\mathbf{E}\left( t \right)=\left[ E_{CEI}\left( t \right),E_{RES}\left( t \right),E_{EER}\left( t \right),E_{GIR}\left( t \right) \right]^{T}$$

where:

- $E_{CEI}(t)$: Carbon emission intensity
- $E_{RES}(t)$: Renewable energy share
- $E_{EER}(t)$: Energy efficiency rating
- $E_{GIR}(t)$: Green investment ratio

## C.4 Market Condition Factors

$$\mathbf{M}\left( t \right)=\left[ M_{MCI}\left( t \right),M_{OCR}\left( t \right),M_{TAR}\left( t \right) \right]^{T}$$

where:

- $M_{MCI}(t)$: Market competition index
- $M_{OCR}(t)$: Operational cost ratio
- $M_{TAR}(t)$: Technology adoption rate

## C.5 Hierarchical Decomposition

The policy effect is decomposed into direct and indirect components:

$$PE\left( t \right)=PE_{direct}\left( t \right)+PE_{indirect}\left( t \right)+PE_{interaction}\left( t \right)$$

The direct effect captures immediate policy impacts:

$$PE_{direct}\left( t \right)=\sum_{i=1}^{8} \beta_{i}P_{i}\left( t \right)$$

The indirect effect captures mediated pathways:

$$PE_{indirect}\left( t \right)=\sum_{i=1}^{8} \sum_{j=1}^{4} \gamma_{ij}P_{i}\left( t \right)\cdot E_{j}\left( t \right)$$

The interaction effect captures policy synergies:

$$PE_{interaction}\left( t \right)=\sum_{i=1}^{8} \sum_{k>i}^{8} \delta_{ik}P_{i}\left( t \right)\cdot P_{k}\left( t \right)$$

# Supplementary Appendix D: Optimization Algorithm Specifications

This appendix provides the complete mathematical formulations for parameter updates and learning rate scheduling referenced in Section III.3 of the main text.

## D.1 Adam Optimizer Parameter Updates

The parameter update equations for the variational mean parameters follow:

$$m_{t}^{\left( \mu\right)}=\beta_{1}m_{t-1}^{\left( \mu\right)}+\left( 1-\beta_{1} \right)g_{t}^{\left( \mu\right)}$$

$$v_{t}^{\left( \mu\right)}=\beta_{2}v_{t-1}^{\left( \mu\right)}+\left( 1-\beta_{2} \right)\left( g_{t}^{\left( \mu\right)} \right)^{2}$$

where:

- $m_{t}^{\left( \mu\right)}$and $v_{t}^{\left( \mu\right)}$represent the first and second moment estimates for mean parameters
- $g_{t}^{\left( \mu\right)}$denotes the gradient with respect to mean parameters
- $\beta_{1}=0.9$, $\beta_{2}=0.999$are exponential decay rates

Bias-corrected estimates:

$$\hat{m}_{t}^{\left( \mu\right)}=\frac{m_{t}^{\left( \mu\right)}}{1-\beta_{1}^{t}}$$

$$\hat{v}_{t}^{\left( \mu\right)}=\frac{v_{t}^{\left( \mu\right)}}{1-\beta_{2}^{t}}$$

Parameter update rule:

$$\mu_{t+1}=\mu_{t}-\eta\cdot\frac{\hat{m}_{t}^{\left( \mu\right)}}{\sqrt{\hat{v}_{t}^{\left( \mu\right)}}+\epsilon}$$

where $\epsilon={10}^{-8}$ ensures numerical stability.

## D.2 Separate Learning Rates for Variance Parameters

For variance parameters, we employ a reduced learning rate:

$$\eta_{\sigma}=0.1\cdot\eta_{\mu}$$

This slower update rate for variance parameters prevents premature collapse of the posterior and maintains meaningful uncertainty estimates throughout training.

## D.3 Adaptive Learning Rate Schedule

The adaptive learning rate formula integrates multiple feedback mechanisms:

$$\eta_{t}=\eta_{0}\cdot min\left( \frac{1}{\sqrt{t}},cos\left( \frac{\pi t}{2T} \right),\frac{1}{1+\lambda\cdot\text{plateau}_{t}} \right)$$

where:

- $\eta_{0}=0.003$represents the initial learning rate
- T denotes the total training epochs
- $\lambda=0.1$controls plateau sensitivity
- $\text{plateau}_{t}$measures performance stagnation periods

## D.4 Cosine Annealing Component

The cosine annealing schedule provides smooth learning rate decay:

$$\eta_{t}^{cos}=\eta_{min}+\frac{1}{2}\left( \eta_{0}-\eta_{min} \right)\left( 1+cos\left( \frac{t\cdot\pi}{T} \right) \right)$$

where $\eta_{min}=0.0001$ is the minimum learning rate.

## D.5 Plateau Detection and Response

The plateau detection mechanism monitors validation loss over a window of $w=10$ epochs:

$$\text{plateau}_{t}=\left\{ \begin{matrix} \text{plateau}_{t-1}+1 & \text{if }\mathcal{L}_{val}\left( t \right)\geq\min_{i\in\left[ t-w,t-1 \right]}\mathcal{L}_{val}\left( i \right) \\ 0 & \text{otherwise} \end{matrix} \right.$$

## D.6 Gradient Clipping

To prevent exploding gradients, we apply norm-based gradient clipping:

$$\hat{g}=\left\{ \begin{matrix} g & \text{if }\parallel g\parallel\leq\tau\\ \tau\cdot\frac{g}{\parallel g\parallel} & \text{otherwise} \end{matrix} \right.$$

where $\tau=1.0$ is the clipping threshold.

# Supplementary Appendix E: Additional Tables

## Table S1. Hyperparameter Search Space and Optimal Configuration

| Hyperparameter | Search Range | Search Method | Optimal Value | Sensitivity |
| --- | --- | --- | --- | --- |
| Initial Learning Rate | [1e-4, 1e-2] | Log-uniform | 0.003 | High |
| Learning Rate Decay | [0.9, 0.99] | Uniform | 0.95 | Medium |
| Batch Size | [32, 128] | Discrete | 64 | Low |
| Dropout Rate | [0.1, 0.5] | Uniform | 0.2 | Medium |
| KL Divergence Weight | [0.1, 1.0] | Uniform | 0.3 | High |
| Prior Precision (α) | [0.1, 10.0] | Log-uniform | 1.0 | Medium |
| Monte Carlo Samples | [10, 100] | Discrete | 50 | Low |
| Hidden Layer Width | [32, 256] | Discrete | 128 | Medium |
| Number of Hidden Layers | [3, 8] | Discrete | 5 | Medium |
| Activation Function | ReLU, ELU, SELU | Categorical | ReLU | Low |
| Weight Initialization | Xavier, He, Normal | Categorical | Xavier Normal | Low |
| Optimizer | Adam, AdamW, SGD | Categorical | Adam | Medium |
| Gradient Clip Norm | [0.5, 5.0] | Uniform | 1.0 | Low |
| Early Stopping Patience | [10, 50] | Discrete | 20 | Low |
| Validation Split Ratio | [0.1, 0.2] | Uniform | 0.15 | Low |

## Table S2. Complete Data Source Description

| Data Category | Source Institution | Time Coverage | Spatial Coverage | Update Frequency | Data Format |
| --- | --- | --- | --- | --- | --- |
| Carbon Emission Data | Fujian Provincial Department of Ecology and Environment | 2018-2024 | 9 prefectures | Monthly | Excel/CSV |
| Renewable Energy Statistics | National Energy Administration Fujian Branch | 2018-2024 | Provincial | Quarterly | PDF/Excel |
| Power Generation Data | State Grid Fujian Electric Power Co. | 2018-2024 | 67 utilities | Daily aggregated to monthly | Database |
| Policy Implementation Records | Fujian Development and Reform Commission | 2018-2024 | Provincial | Event-based | PDF |
| Economic Indicators | Fujian Provincial Bureau of Statistics | 2018-2024 | 9 prefectures | Monthly/Quarterly | Excel |
| Environmental Investment | Enterprise Annual Reports | 2018-2024 | 67 utilities | Annual | PDF |
| Customer Satisfaction | Third-party Survey Agency | 2019-2024 | Provincial | Semi-annual | Excel |
| System Reliability | State Grid Dispatch Center | 2018-2024 | 9 prefectures | Monthly | Database |
| Technology Adoption | Enterprise Technology Reports | 2018-2024 | 67 utilities | Annual | PDF/Excel |
| Regulatory Compliance | Fujian Energy Regulatory Office | 2018-2024 | 67 utilities | Quarterly | PDF |

## Table S3. Variable Definitions and Measurement Methods

| Variable | Definition | Unit | Measurement Method | Data Transformation |
| --- | --- | --- | --- | --- |
| Carbon Emission Intensity | CO2 emissions per unit electricity generated | kg CO2/kWh | Direct measurement from emission monitoring systems | Log transformation |
| Renewable Energy Share | Proportion of electricity from renewable sources | Percentage | Generation capacity weighted calculation | Logit transformation |
| Policy Intensity Index | Composite measure of policy stringency | Index (1-10) | Weighted average of 12 policy indicators | Min-max normalization |
| Energy Efficiency Rating | Technical efficiency of power generation | Dimensionless | Data envelopment analysis | Standardization |
| Green Investment Ratio | Environmental investment as proportion of total | Percentage | Accounting records analysis | Square root transformation |
| Compliance Score | Degree of regulatory compliance | Score (0-100) | Weighted checklist evaluation | None |
| Market Competition Index | Herfindahl-Hirschman Index inverse | Index | Market share calculation | Log transformation |
| Customer Satisfaction | Survey-based satisfaction measure | Score (1-10) | Stratified random sampling survey | None |
| Operational Cost Ratio | Operating costs per unit revenue | Percentage | Financial statement analysis | Logit transformation |
| Technology Adoption Rate | New technology implementation level | Percentage | Technology inventory assessment | Logit transformation |

## Table S4. Correlation Matrix of Key Variables

| Variable | CEI | RES | PII | EER | GIR | CS | MCI | CuS | OCR | TAR |
| --- | --- | --- | --- | --- | --- | --- | --- | --- | --- | --- |
| Carbon Emission Intensity (CEI) | 1.000 | -0.672 | -0.584 | -0.523 | -0.456 | -0.398 | 0.234 | -0.312 | 0.445 | -0.389 |
| Renewable Energy Share (RES) | -0.672 | 1.000 | 0.623 | 0.567 | 0.534 | 0.478 | -0.189 | 0.423 | -0.378 | 0.512 |
| Policy Intensity Index (PII) | -0.584 | 0.623 | 1.000 | 0.489 | 0.612 | 0.534 | -0.267 | 0.356 | -0.289 | 0.478 |
| Energy Efficiency Rating (EER) | -0.523 | 0.567 | 0.489 | 1.000 | 0.423 | 0.512 | -0.145 | 0.489 | -0.456 | 0.534 |
| Green Investment Ratio (GIR) | -0.456 | 0.534 | 0.612 | 0.423 | 1.000 | 0.445 | -0.178 | 0.312 | -0.234 | 0.456 |
| Compliance Score (CS) | -0.398 | 0.478 | 0.534 | 0.512 | 0.445 | 1.000 | -0.123 | 0.534 | -0.312 | 0.389 |
| Market Competition Index (MCI) | 0.234 | -0.189 | -0.267 | -0.145 | -0.178 | -0.123 | 1.000 | 0.145 | 0.289 | -0.134 |
| Customer Satisfaction (CuS) | -0.312 | 0.423 | 0.356 | 0.489 | 0.312 | 0.534 | 0.145 | 1.000 | -0.267 | 0.345 |
| Operational Cost Ratio (OCR) | 0.445 | -0.378 | -0.289 | -0.456 | -0.234 | -0.312 | 0.289 | -0.267 | 1.000 | -0.278 |
| Technology Adoption Rate (TAR) | -0.389 | 0.512 | 0.478 | 0.534 | 0.456 | 0.389 | -0.134 | 0.345 | -0.278 | 1.000 |

Note: All correlations with absolute value greater than 0.15 are statistically significant at p < 0.05.

## Table S5. Missing Data Patterns and Imputation Summary

| Variable | Total Observations | Missing Count | Missing Percentage | Missing Pattern | Imputation Method | Imputation Quality (R²) |
| --- | --- | --- | --- | --- | --- | --- |
| Carbon Emission Intensity | 1248 | 23 | 1.84% | MCAR | EM Algorithm | 0.956 |
| Renewable Energy Share | 1248 | 18 | 1.44% | MCAR | EM Algorithm | 0.967 |
| Policy Intensity Index | 1248 | 0 | 0.00% | Complete | N/A | N/A |
| Energy Efficiency Rating | 1248 | 45 | 3.61% | MAR | Multiple Imputation | 0.923 |
| Green Investment Ratio | 1248 | 67 | 5.37% | MAR | Multiple Imputation | 0.912 |
| Compliance Score | 1248 | 12 | 0.96% | MCAR | EM Algorithm | 0.978 |
| Market Competition Index | 1248 | 8 | 0.64% | MCAR | Mean Imputation | 0.989 |
| Customer Satisfaction | 1248 | 156 | 12.50% | MAR | Multiple Imputation | 0.867 |
| Operational Cost Ratio | 1248 | 34 | 2.72% | MAR | EM Algorithm | 0.945 |
| Technology Adoption Rate | 1248 | 89 | 7.13% | MAR | Multiple Imputation | 0.891 |

Note: MCAR = Missing Completely at Random; MAR = Missing at Random. Imputation quality measured by cross-validation R² on observed values.

## Table S6. Detailed Scenario Analysis Results with Confidence Intervals

| Scenario | Policy Intensity | Environmental Effect | 90% CI | 95% CI | 99% CI | Economic Cost Index | Net Benefit Score |
| --- | --- | --- | --- | --- | --- | --- | --- |
| S1: Baseline | 2.0 | 0.285 | [0.271, 0.299] | [0.267, 0.303] | [0.258, 0.312] | 0.142 | 0.143 |
| S2: Minimal Intervention | 2.5 | 0.328 | [0.312, 0.344] | [0.307, 0.349] | [0.297, 0.359] | 0.178 | 0.150 |
| S3: Low Intensity | 3.5 | 0.412 | [0.394, 0.430] | [0.389, 0.435] | [0.378, 0.446] | 0.285 | 0.127 |
| S4: Low-Medium | 4.5 | 0.523 | [0.502, 0.544] | [0.496, 0.550] | [0.483, 0.563] | 0.398 | 0.125 |
| S5: Medium | 5.5 | 0.634 | [0.612, 0.656] | [0.607, 0.661] | [0.593, 0.675] | 0.512 | 0.122 |
| S6: Medium-High | 6.5 | 0.728 | [0.704, 0.752] | [0.699, 0.757] | [0.684, 0.772] | 0.623 | 0.105 |
| S7: High | 7.5 | 0.803 | [0.778, 0.828] | [0.772, 0.834] | [0.756, 0.850] | 0.734 | 0.069 |
| S8: Very High | 8.5 | 0.859 | [0.832, 0.886] | [0.826, 0.892] | [0.809, 0.909] | 0.845 | 0.014 |
| S9: Intensive | 9.5 | 0.894 | [0.863, 0.925] | [0.856, 0.932] | [0.838, 0.950] | 0.956 | -0.062 |
| S10: Maximum | 10.0 | 0.912 | [0.879, 0.945] | [0.873, 0.951] | [0.854, 0.970] | 1.000 | -0.088 |

Note: Net Benefit Score = (Environmental Effect - Economic Cost Index) normalized to [-1, 1] range.

## Table S7. Policy Instrument Effectiveness Decomposition

| Policy Instrument | Direct Effect | Indirect Effect | Total Effect | Standard Error | 95% CI | Effect Ranking |
| --- | --- | --- | --- | --- | --- | --- |
| Renewable Energy Subsidies | 0.187 | 0.095 | 0.282 | 0.034 | [0.215, 0.349] | 1 |
| Carbon Pricing Mechanism | 0.156 | 0.078 | 0.234 | 0.029 | [0.177, 0.291] | 2 |
| Regulatory Enforcement | 0.134 | 0.056 | 0.190 | 0.025 | [0.141, 0.239] | 3 |
| Energy Efficiency Standards | 0.098 | 0.045 | 0.143 | 0.022 | [0.100, 0.186] | 4 |
| Technology R&D Incentives | 0.067 | 0.034 | 0.101 | 0.018 | [0.066, 0.136] | 5 |
| Green Procurement Policies | 0.045 | 0.023 | 0.068 | 0.015 | [0.039, 0.097] | 6 |
| Information Disclosure Requirements | 0.034 | 0.018 | 0.052 | 0.012 | [0.029, 0.075] | 7 |
| Stakeholder Engagement Programs | 0.023 | 0.012 | 0.035 | 0.009 | [0.017, 0.053] | 8 |

Note: Effects measured as standardized regression coefficients. Indirect effects calculated through mediation analysis.

## Table S8. Temporal Lag Analysis Results

| Variable | Lag 0 Effect | Lag 1 Quarter | Lag 2 Quarters | Lag 3 Quarters | Lag 4 Quarters | Cumulative Effect | Peak Lag |
| --- | --- | --- | --- | --- | --- | --- | --- |
| Renewable Energy Subsidies | 0.089 | 0.112 | 0.067 | 0.034 | 0.012 | 0.314 | 1 |
| Carbon Pricing | 0.045 | 0.078 | 0.089 | 0.056 | 0.023 | 0.291 | 2 |
| Regulatory Enforcement | 0.112 | 0.067 | 0.034 | 0.015 | 0.006 | 0.234 | 0 |
| Efficiency Standards | 0.023 | 0.045 | 0.056 | 0.045 | 0.023 | 0.192 | 2 |
| R&D Incentives | 0.012 | 0.023 | 0.034 | 0.045 | 0.034 | 0.148 | 3 |
| Green Procurement | 0.034 | 0.034 | 0.023 | 0.012 | 0.006 | 0.109 | 0-1 |

Note: Effects measured as partial regression coefficients. Peak Lag indicates the quarter with maximum effect.

## Table S9. Model Convergence Diagnostics

| Diagnostic Metric | Value | Threshold | Status | Interpretation |
| --- | --- | --- | --- | --- |
| Training Loss (Final) | 0.0234 | < 0.05 | Pass | Good convergence |
| Validation Loss (Final) | 0.0267 | < 0.06 | Pass | Good generalization |
| Training-Validation Gap | 0.0033 | < 0.02 | Pass | No overfitting |
| Gradient Norm (Mean) | 0.0089 | < 0.1 | Pass | Stable gradients |
| Gradient Norm (Max) | 0.0456 | < 1.0 | Pass | No exploding gradients |
| ELBO Convergence Rate | 0.9987 | > 0.99 | Pass | Convergent |
| KL Divergence (Final) | 1.234 | < 5.0 | Pass | Reasonable posterior |
| Effective Sample Size | 847 | > 100 | Pass | Sufficient samples |
| Gelman-Rubin R-hat | 1.003 | < 1.1 | Pass | Chain convergence |
| Autocorrelation (Lag 1) | 0.087 | < 0.3 | Pass | Low autocorrelation |

## Table S10. Sensitivity Analysis: Prior Specification

| Prior Configuration | Prior Mean | Prior Variance | Posterior Mean | Posterior Variance | ELBO | Prediction R² |
| --- | --- | --- | --- | --- | --- | --- |
| Uninformative | 0.0 | 10.0 | 0.234 | 0.089 | -456.7 | 0.878 |
| Weakly Informative | 0.0 | 1.0 | 0.228 | 0.067 | -423.4 | 0.892 |
| Moderately Informative | 0.0 | 0.5 | 0.225 | 0.056 | -412.8 | 0.889 |
| Strongly Informative | 0.0 | 0.1 | 0.218 | 0.034 | -398.2 | 0.871 |
| Domain-Specific | 0.1 | 0.5 | 0.231 | 0.058 | -408.5 | 0.895 |

Note: Domain-Specific prior incorporates information from literature on typical policy effect magnitudes.

## Table S11. Cross-Validation Results by Temporal Fold

| Fold | Training Period | Validation Period | Training MSE | Validation MSE | Training R² | Validation R² | ECE |
| --- | --- | --- | --- | --- | --- | --- | --- |
| 1 | 2018-2020 | 2021 | 0.0223 | 0.0278 | 0.903 | 0.879 | 0.0867 |
| 2 | 2018-2021 | 2022 | 0.0218 | 0.0265 | 0.908 | 0.885 | 0.0823 |
| 3 | 2018-2022 | 2023 | 0.0215 | 0.0256 | 0.912 | 0.891 | 0.0798 |
| 4 | 2018-2023 | 2024 | 0.0212 | 0.0247 | 0.915 | 0.897 | 0.0789 |
| Average | — | — | 0.0217 | 0.0262 | 0.910 | 0.888 | 0.0819 |
| Std Dev | — | — | 0.0005 | 0.0013 | 0.005 | 0.008 | 0.0035 |

## Table S12. Regional Heterogeneity Analysis

| Prefecture | Sample Size | Mean Policy Intensity | Mean Effect | Model R² | Regional Fixed Effect | Standard Error |
| --- | --- | --- | --- | --- | --- | --- |
| Fuzhou | 187 | 4.23 | 0.567 | 0.912 | 0.034 | 0.012 |
| Xiamen | 156 | 4.67 | 0.623 | 0.923 | 0.078 | 0.015 |
| Quanzhou | 178 | 3.89 | 0.512 | 0.897 | -0.023 | 0.014 |
| Zhangzhou | 134 | 3.56 | 0.478 | 0.878 | -0.056 | 0.018 |
| Nanping | 112 | 3.12 | 0.423 | 0.856 | -0.089 | 0.021 |
| Longyan | 123 | 3.34 | 0.445 | 0.867 | -0.067 | 0.019 |
| Sanming | 118 | 3.23 | 0.434 | 0.861 | -0.078 | 0.020 |
| Ningde | 128 | 3.78 | 0.489 | 0.889 | -0.034 | 0.017 |
| Putian | 112 | 3.45 | 0.456 | 0.872 | -0.045 | 0.019 |

Note: Regional fixed effects estimated relative to provincial mean; positive values indicate above-average policy responsiveness.

## Table S13. Uncertainty Decomposition by Scenario

| Scenario | Total Variance | Aleatoric Uncertainty | Epistemic Uncertainty | Aleatoric Share | Epistemic Share |
| --- | --- | --- | --- | --- | --- |
| S1: Baseline | 0.00089 | 0.00067 | 0.00022 | 75.3% | 24.7% |
| S3: Low Intensity | 0.00134 | 0.00089 | 0.00045 | 66.4% | 33.6% |
| S5: Medium | 0.00178 | 0.00112 | 0.00066 | 62.9% | 37.1% |
| S7: High | 0.00234 | 0.00134 | 0.00100 | 57.3% | 42.7% |
| S10: Maximum | 0.00389 | 0.00178 | 0.00211 | 45.8% | 54.2% |

Note: Higher epistemic share at extreme scenarios indicates greater model uncertainty due to sparse training data in those regions.

## Table S14. Computational Performance Metrics

| Metric | Value | Hardware Configuration |
| --- | --- | --- |
| Training Time (Total) | 45.3 minutes | NVIDIA RTX 3090, 24GB VRAM |
| Training Time per Epoch | 5.4 seconds | Intel i9-12900K, 64GB RAM |
| Inference Time (Single Sample) | 0.23 ms | Python 3.9, PyTorch 2.0 |
| Inference Time (Batch of 100) | 4.7 ms | CUDA 11.8 |
| Memory Usage (Training) | 8.4 GB | Batch size 64 |
| Memory Usage (Inference) | 1.2 GB | 50 MC samples |
| Model Parameters (Total) | 89,745 | 5 hidden layers |
| Variational Parameters | 179,490 | Mean + variance for each weight |

# Supplementary Appendix F: Python Implementation Code

"""
Bayesian Neural Network for Policy Effect Prediction
Implementation for Green Transformation of Power Business Environment
"""

import numpy as np
import pandas as pd
import torch
import torch.nn as nn
import torch.nn.functional as F
from torch.utils.data import DataLoader, TensorDataset
from sklearn.preprocessing import StandardScaler
from sklearn.model_selection import TimeSeriesSplit
import warnings
warnings.filterwarnings('ignore')

# Set random seeds for reproducibility
RANDOM_SEED = 42
np.random.seed(RANDOM_SEED)
torch.manual_seed(RANDOM_SEED)
if torch.cuda.is_available():
 torch.cuda.manual_seed(RANDOM_SEED)

# Device configuration
device = torch.device('cuda' if torch.cuda.is_available() else 'cpu')


class BayesianLinear(nn.Module):
 """
 Bayesian Linear Layer with variational inference
 Implements weight uncertainty using Gaussian distributions
 """

 def __init__(self, in_features, out_features, prior_var=1.0):
 super(BayesianLinear, self).__init__()
 self.in_features = in_features
 self.out_features = out_features
 self.prior_var = prior_var

 # Variational parameters for weights (mean and log variance)
 self.weight_mu = nn.Parameter(
 torch.Tensor(out_features, in_features).normal_(0, 0.02)
 )
 self.weight_log_var = nn.Parameter(
 torch.Tensor(out_features, in_features).fill_(-5)
 )

 # Variational parameters for bias
 self.bias_mu = nn.Parameter(
 torch.Tensor(out_features).normal_(0, 0.02)
 )
 self.bias_log_var = nn.Parameter(
 torch.Tensor(out_features).fill_(-5)
 )

 # Prior parameters
 self.prior_weight_mu = 0
 self.prior_weight_var = prior_var
 self.prior_bias_mu = 0
 self.prior_bias_var = prior_var

 def forward(self, x, sample=True):
 if sample:
 # Reparameterization trick
 weight_var = torch.exp(self.weight_log_var)
 weight_std = torch.sqrt(weight_var)
 weight_epsilon = torch.randn_like(weight_std)
 weight = self.weight_mu + weight_std * weight_epsilon

 bias_var = torch.exp(self.bias_log_var)
 bias_std = torch.sqrt(bias_var)
 bias_epsilon = torch.randn_like(bias_std)
 bias = self.bias_mu + bias_std * bias_epsilon
 else:
 weight = self.weight_mu
 bias = self.bias_mu

 return F.linear(x, weight, bias)

 def kl_divergence(self):
 """
 Compute KL divergence between posterior and prior
 KL[q(w)||p(w)] for Gaussian distributions
 """
 weight_var = torch.exp(self.weight_log_var)
 bias_var = torch.exp(self.bias_log_var)

 # KL for weights
 kl_weight = 0.5 * torch.sum(
 weight_var / self.prior_weight_var +
 (self.weight_mu - self.prior_weight_mu) ** 2 / self.prior_weight_var -
 1 + np.log(self.prior_weight_var) - self.weight_log_var
 )

 # KL for bias
 kl_bias = 0.5 * torch.sum(
 bias_var / self.prior_bias_var +
 (self.bias_mu - self.prior_bias_mu) ** 2 / self.prior_bias_var -
 1 + np.log(self.prior_bias_var) - self.bias_log_var
 )

 return kl_weight + kl_bias


class BayesianNeuralNetwork(nn.Module):
 """
 Bayesian Neural Network for Policy Effect Prediction
 Architecture: Input -> Hidden Layers (128-96-64-32-16) -> Output
 """

 def __init__(self, input_dim, hidden_dims=[128, 96, 64, 32, 16],
 output_dim=1, prior_var=1.0, dropout_rate=0.2):
 super(BayesianNeuralNetwork, self).__init__()

 self.input_dim = input_dim
 self.hidden_dims = hidden_dims
 self.output_dim = output_dim
 self.dropout_rate = dropout_rate

 # Build network layers
 layers = []
 prev_dim = input_dim

 for hidden_dim in hidden_dims:
 layers.append(BayesianLinear(prev_dim, hidden_dim, prior_var))
 layers.append(nn.ReLU())
 layers.append(nn.Dropout(dropout_rate))
 prev_dim = hidden_dim

 # Output layer (mean prediction)
 layers.append(BayesianLinear(prev_dim, output_dim, prior_var))

 self.layers = nn.ModuleList(layers)

 # Learnable noise variance for aleatoric uncertainty
 self.log_noise_var = nn.Parameter(torch.tensor(-3.0))

 def forward(self, x, sample=True):
 for layer in self.layers:
 if isinstance(layer, BayesianLinear):
 x = layer(x, sample=sample)
 else:
 x = layer(x)
 return x

 def kl_divergence(self):
 """Total KL divergence across all Bayesian layers"""
 kl = 0
 for layer in self.layers:
 if isinstance(layer, BayesianLinear):
 kl += layer.kl_divergence()
 return kl

 def predict_with_uncertainty(self, x, num_samples=50):
 """
 Monte Carlo prediction with uncertainty quantification
 Returns mean, aleatoric uncertainty, and epistemic uncertainty
 """
 self.eval()
 predictions = []

 with torch.no_grad():
 for _ in range(num_samples):
 pred = self.forward(x, sample=True)
 predictions.append(pred)

 predictions = torch.stack(predictions)

 # Mean prediction
 mean_pred = predictions.mean(dim=0)

 # Epistemic uncertainty (model uncertainty)
 epistemic_var = predictions.var(dim=0)

 # Aleatoric uncertainty (data noise)
 aleatoric_var = torch.exp(self.log_noise_var)

 # Total uncertainty
 total_var = epistemic_var + aleatoric_var

 return mean_pred, epistemic_var, aleatoric_var, total_var


class PolicyEffectPredictor:
 """
 Complete pipeline for Bayesian policy effect prediction
 Includes data preprocessing, training, and evaluation
 """

 def __init__(self, config=None):
 self.config = config or self._default_config()
 self.model = None
 self.scaler_X = StandardScaler()
 self.scaler_y = StandardScaler()
 self.training_history = []

 def _default_config(self):
 return {
 'hidden_dims': [128, 96, 64, 32, 16],
 'prior_var': 1.0,
 'dropout_rate': 0.2,
 'learning_rate': 0.003,
 'batch_size': 64,
 'epochs': 500,
 'kl_weight': 0.3,
 'early_stopping_patience': 20,
 'mc_samples': 50,
 'validation_split': 0.15
 }

 def preprocess_data(self, X, y, fit=True):
 """Standardize features and target"""
 if fit:
 X_scaled = self.scaler_X.fit_transform(X)
 y_scaled = self.scaler_y.fit_transform(y.reshape(-1, 1))
 else:
 X_scaled = self.scaler_X.transform(X)
 y_scaled = self.scaler_y.transform(y.reshape(-1, 1))

 return X_scaled, y_scaled.flatten()

 def build_model(self, input_dim):
 """Initialize the Bayesian Neural Network"""
 self.model = BayesianNeuralNetwork(
 input_dim=input_dim,
 hidden_dims=self.config['hidden_dims'],
 prior_var=self.config['prior_var'],
 dropout_rate=self.config['dropout_rate']
 ).to(device)

 return self.model

 def compute_elbo_loss(self, predictions, targets, kl_divergence, num_samples):
 """
 Compute Evidence Lower Bound (ELBO) loss
 ELBO = E[log p(y|x,w)] - KL[q(w)||p(w)]
 """
 # Negative log likelihood (assuming Gaussian)
 noise_var = torch.exp(self.model.log_noise_var)
 nll = 0.5 * torch.log(2 * np.pi * noise_var) + \
 0.5 * ((predictions - targets) ** 2) / noise_var
 nll = nll.mean()

 # KL divergence weighted by factor
 kl_weight = self.config['kl_weight'] / num_samples
 kl_term = kl_weight * kl_divergence

 # Total ELBO loss (negative ELBO for minimization)
 loss = nll + kl_term

 return loss, nll, kl_term

 def train(self, X_train, y_train, X_val=None, y_val=None):
 """
 Train the Bayesian Neural Network using variational inference
 """
 # Preprocess data
 X_train_scaled, y_train_scaled = self.preprocess_data(X_train, y_train, fit=True)

 if X_val is not None:
 X_val_scaled, y_val_scaled = self.preprocess_data(X_val, y_val, fit=False)

 # Convert to tensors
 X_train_tensor = torch.FloatTensor(X_train_scaled).to(device)
 y_train_tensor = torch.FloatTensor(y_train_scaled).to(device)

 # Create data loader
 train_dataset = TensorDataset(X_train_tensor, y_train_tensor)
 train_loader = DataLoader(
 train_dataset,
 batch_size=self.config['batch_size'],
 shuffle=True
 )

 # Build model
 input_dim = X_train.shape[1]
 self.build_model(input_dim)

 # Optimizer with separate learning rates
 optimizer = torch.optim.Adam([
 {'params': [p for n, p in self.model.named_parameters() if 'mu' in n],
 'lr': self.config['learning_rate']},
 {'params': [p for n, p in self.model.named_parameters() if 'log_var' in n],
 'lr': self.config['learning_rate'] * 0.1}
 ])

 # Learning rate scheduler
 scheduler = torch.optim.lr_scheduler.CosineAnnealingLR(
 optimizer, T_max=self.config['epochs']
 )

 # Training loop
 best_val_loss = float('inf')
 patience_counter = 0
 num_samples = len(train_loader.dataset)

 for epoch in range(self.config['epochs']):
 self.model.train()
 train_loss = 0
 train_nll = 0
 train_kl = 0

 for batch_X, batch_y in train_loader:
 optimizer.zero_grad()

 # Forward pass with sampling
 predictions = self.model(batch_X, sample=True)

 # Compute ELBO loss
 kl_div = self.model.kl_divergence()
 loss, nll, kl = self.compute_elbo_loss(
 predictions.squeeze(), batch_y, kl_div, num_samples
 )

 # Backward pass
 loss.backward()

 # Gradient clipping
 torch.nn.utils.clip_grad_norm_(self.model.parameters(), max_norm=1.0)

 optimizer.step()

 train_loss += loss.item()
 train_nll += nll.item()
 train_kl += kl.item()

 # Average losses
 train_loss /= len(train_loader)
 train_nll /= len(train_loader)
 train_kl /= len(train_loader)

 # Validation
 if X_val is not None:
 val_loss = self._validate(X_val_scaled, y_val_scaled)

 # Early stopping check
 if val_loss < best_val_loss:
 best_val_loss = val_loss
 patience_counter = 0
 # Save best model
 self.best_model_state = self.model.state_dict().copy()
 else:
 patience_counter += 1

 if patience_counter >= self.config['early_stopping_patience']:
 print(f"Early stopping at epoch {epoch+1}")
 break

 scheduler.step()

 # Record history
 self.training_history.append({
 'epoch': epoch + 1,
 'train_loss': train_loss,
 'train_nll': train_nll,
 'train_kl': train_kl,
 'val_loss': val_loss if X_val is not None else None
 })

 # Print progress
 if (epoch + 1) % 50 == 0:
 print(f"Epoch {epoch+1}/{self.config['epochs']}: "
 f"Train Loss = {train_loss:.4f}, "
 f"Val Loss = {val_loss:.4f}" if X_val is not None else "")

 # Load best model
 if hasattr(self, 'best_model_state'):
 self.model.load_state_dict(self.best_model_state)

 return self.training_history

 def _validate(self, X_val_scaled, y_val_scaled):
 """Compute validation loss"""
 self.model.eval()
 X_val_tensor = torch.FloatTensor(X_val_scaled).to(device)
 y_val_tensor = torch.FloatTensor(y_val_scaled).to(device)

 with torch.no_grad():
 predictions = self.model(X_val_tensor, sample=False)
 mse_loss = F.mse_loss(predictions.squeeze(), y_val_tensor)

 return mse_loss.item()

 def predict(self, X, return_uncertainty=True):
 """
 Make predictions with uncertainty quantification
 """
 X_scaled = self.scaler_X.transform(X)
 X_tensor = torch.FloatTensor(X_scaled).to(device)

 mean_pred, epistemic_var, aleatoric_var, total_var = \
 self.model.predict_with_uncertainty(
 X_tensor,
 num_samples=self.config['mc_samples']
 )

 # Transform predictions back to original scale
 mean_pred_np = mean_pred.cpu().numpy()
 mean_pred_original = self.scaler_y.inverse_transform(
 mean_pred_np.reshape(-1, 1)
 ).flatten()

 # Scale variances
 scale_factor = self.scaler_y.scale_[0] ** 2
 epistemic_var_original = epistemic_var.cpu().numpy() * scale_factor
 aleatoric_var_original = aleatoric_var.item() * scale_factor
 total_var_original = total_var.cpu().numpy() * scale_factor

 if return_uncertainty:
 return {
 'mean': mean_pred_original,
 'epistemic_uncertainty': np.sqrt(epistemic_var_original),
 'aleatoric_uncertainty': np.sqrt(aleatoric_var_original),
 'total_uncertainty': np.sqrt(total_var_original),
 'confidence_interval_95': (
 mean_pred_original - 1.96 * np.sqrt(total_var_original),
 mean_pred_original + 1.96 * np.sqrt(total_var_original)
 )
 }
 else:
 return mean_pred_original

 def evaluate(self, X_test, y_test):
 """
 Comprehensive model evaluation
 """
 predictions = self.predict(X_test, return_uncertainty=True)
 y_pred = predictions['mean']

 # Compute metrics
 mse = np.mean((y_test - y_pred) ** 2)
 mae = np.mean(np.abs(y_test - y_pred))
 rmse = np.sqrt(mse)

 # R-squared
 ss_res = np.sum((y_test - y_pred) ** 2)
 ss_tot = np.sum((y_test - np.mean(y_test)) ** 2)
 r2 = 1 - (ss_res / ss_tot)

 # Prediction interval coverage
 ci_lower, ci_upper = predictions['confidence_interval_95']
 coverage = np.mean((y_test >= ci_lower) & (y_test <= ci_upper))

 # Expected Calibration Error
 ece = self._compute_ece(y_test, y_pred, predictions['total_uncertainty'])

 return {
 'MSE': mse,
 'MAE': mae,
 'RMSE': rmse,
 'R2': r2,
 'Coverage_95': coverage,
 'ECE': ece
 }

 def _compute_ece(self, y_true, y_pred, uncertainties, n_bins=10):
 """Compute Expected Calibration Error"""
 # Sort by uncertainty
 sorted_indices = np.argsort(uncertainties.flatten())

 # Create bins
 bin_size = len(y_true) // n_bins
 ece = 0

 for i in range(n_bins):
 start_idx = i * bin_size
 end_idx = (i + 1) * bin_size if i < n_bins - 1 else len(y_true)

 bin_indices = sorted_indices[start_idx:end_idx]
 bin_errors = np.abs(y_true[bin_indices] - y_pred[bin_indices])
 bin_uncertainties = uncertainties.flatten()[bin_indices]

 avg_error = np.mean(bin_errors)
 avg_uncertainty = np.mean(bin_uncertainties)

 ece += (len(bin_indices) / len(y_true)) * np.abs(avg_error - avg_uncertainty)

 return ece


def scenario_analysis(predictor, base_features, intensity_range=np.linspace(2, 10, 9)):
 """
 Conduct policy scenario analysis across different intensity levels
 """
 results = []

 for intensity in intensity_range:
 # Modify policy intensity in features
 scenario_features = base_features.copy()
 scenario_features[:, 0] = intensity # Assuming first feature is policy intensity

 # Get predictions with uncertainty
 predictions = predictor.predict(scenario_features, return_uncertainty=True)

 results.append({
 'intensity': intensity,
 'mean_effect': np.mean(predictions['mean']),
 'std_effect': np.std(predictions['mean']),
 'epistemic_uncertainty': np.mean(predictions['epistemic_uncertainty']),
 'aleatoric_uncertainty': predictions['aleatoric_uncertainty'],
 'ci_lower': np.mean(predictions['confidence_interval_95'][0]),
 'ci_upper': np.mean(predictions['confidence_interval_95'][1])
 })

 return pd.DataFrame(results)


def compute_shap_values(predictor, X, num_samples=100):
 """
 Compute SHAP-like feature importance using Monte Carlo sampling
 """
 n_features = X.shape[1]
 feature_importance = np.zeros(n_features)

 baseline_pred = predictor.predict(X, return_uncertainty=False).mean()

 for i in range(n_features):
 # Permutation-based importance
 importance_samples = []

 for _ in range(num_samples):
 X_permuted = X.copy()
 X_permuted[:, i] = np.random.permutation(X_permuted[:, i])

 permuted_pred = predictor.predict(X_permuted, return_uncertainty=False).mean()
 importance_samples.append(np.abs(baseline_pred - permuted_pred))

 feature_importance[i] = np.mean(importance_samples)

 # Normalize
 feature_importance = feature_importance / feature_importance.sum()

 return feature_importance


# Example usage and main execution
if __name__ == "__main__":
 # Generate synthetic data for demonstration
 np.random.seed(42)
 n_samples = 1248
 n_features = 15

 # Simulate policy data
 X = np.random.randn(n_samples, n_features)
 X[:, 0] = np.random.uniform(1, 10, n_samples) # Policy intensity

 # Non-linear relationship with noise
 y = (0.3 * np.log(X[:, 0] + 1) +
 0.2 * X[:, 1] +
 0.15 * X[:, 2] * X[:, 3] +
 0.1 * np.sin(X[:, 4]) +
 0.05 * np.random.randn(n_samples))

 # Train-test split (temporal)
 split_idx = int(0.8 * n_samples)
 X_train, X_test = X[:split_idx], X[split_idx:]
 y_train, y_test = y[:split_idx], y[split_idx:]

 # Validation split
 val_idx = int(0.85 * len(X_train))
 X_train_final, X_val = X_train[:val_idx], X_train[val_idx:]
 y_train_final, y_val = y_train[:val_idx], y_train[val_idx:]

 # Initialize and train predictor
 config = {
 'hidden_dims': [128, 96, 64, 32, 16],
 'prior_var': 1.0,
 'dropout_rate': 0.2,
 'learning_rate': 0.003,
 'batch_size': 64,
 'epochs': 200,
 'kl_weight': 0.3,
 'early_stopping_patience': 20,
 'mc_samples': 50
 }

 predictor = PolicyEffectPredictor(config)
 history = predictor.train(X_train_final, y_train_final, X_val, y_val)

 # Evaluate
 metrics = predictor.evaluate(X_test, y_test)
 print("\nEvaluation Metrics:")
 for metric, value in metrics.items():
 print(f" {metric}: {value:.4f}")

 # Scenario analysis
 print("\nScenario Analysis:")
 scenarios = scenario_analysis(predictor, X_test[:10])
 print(scenarios.to_string(index=False))

 # Feature importance
 print("\nFeature Importance:")
 importance = compute_shap_values(predictor, X_test[:100])
 for i, imp in enumerate(importance):
 print(f" Feature {i+1}: {imp:.4f}")

# Supplementary Appendix G: Model Configuration Files

## G.1 Training Configuration (config.yaml)

# Bayesian Neural Network Configuration
# Policy Effect Prediction Model

model:
 architecture:
 input_dim: 15
 hidden_dims: [128, 96, 64, 32, 16]
 output_dim: 1
 activation: relu
 dropout_rate: 0.2

 bayesian:
 prior_type: gaussian
 prior_mean: 0.0
 prior_variance: 1.0
 posterior_type: mean_field_gaussian

training:
 optimizer:
 type: adam
 learning_rate: 0.003
 beta1: 0.9
 beta2: 0.999
 epsilon: 1e-8
 weight_decay: 0.0001

 scheduler:
 type: cosine_annealing
 T_max: 500
 eta_min: 0.0001

 loss:
 kl_weight: 0.3
 kl_annealing: false

 regularization:
 gradient_clip_norm: 1.0
 early_stopping_patience: 20

 batch_size: 64
 epochs: 500
 validation_split: 0.15

inference:
 mc_samples: 50
 confidence_level: 0.95

data:
 preprocessing:
 normalization: z_score
 missing_value_method: em_algorithm
 outlier_method: iqr
 outlier_threshold: 3.0

 temporal_split:
 train_end: "2022-12-31"
 val_start: "2023-01-01"
 val_end: "2023-06-30"
 test_start: "2023-07-01"

evaluation:
 metrics:
 - mse
 - mae
 - rmse
 - r2
 - coverage_95
 - ece

 cross_validation:
 type: temporal
 n_splits: 4
 gap: 0

hardware:
 device: cuda
 precision: float32
 num_workers: 4
 pin_memory: true

logging:
 level: INFO
 save_frequency: 10
 tensorboard: true
 checkpoint_frequency: 50

random_seed: 42

## G.2 Data Schema (data_schema.json)

{
 "dataset_name": "Fujian Power System Green Transformation",
 "version": "1.0",
 "created_date": "2024-06-15",
 "last_updated": "2024-12-01",
 "total_records": 1248,
 "temporal_coverage": {
 "start_date": "2018-01-01",
 "end_date": "2024-06-30"
 },
 "spatial_coverage": {
 "region": "Fujian Province, China",
 "prefectures": 9,
 "utilities": 67
 },
 "variables": [
 {
 "name": "carbon_emission_intensity",
 "type": "continuous",
 "unit": "kg_CO2_per_kWh",
 "range": [0.298, 0.854],
 "missing_rate": 0.0184,
 "transformation": "log"
 },
 {
 "name": "renewable_energy_share",
 "type": "continuous",
 "unit": "percentage",
 "range": [0.145, 0.521],
 "missing_rate": 0.0144,
 "transformation": "logit"
 },
 {
 "name": "policy_intensity_index",
 "type": "continuous",
 "unit": "index_1_10",
 "range": [1.20, 6.85],
 "missing_rate": 0.0,
 "transformation": "min_max"
 },
 {
 "name": "energy_efficiency_rating",
 "type": "continuous",
 "unit": "dimensionless",
 "range": [1.42, 4.96],
 "missing_rate": 0.0361,
 "transformation": "standardization"
 },
 {
 "name": "green_investment_ratio",
 "type": "continuous",
 "unit": "percentage",
 "range": [0.067, 0.289],
 "missing_rate": 0.0537,
 "transformation": "sqrt"
 },
 {
 "name": "compliance_score",
 "type": "continuous",
 "unit": "score_0_100",
 "range": [45.2, 98.7],
 "missing_rate": 0.0096,
 "transformation": "none"
 },
 {
 "name": "market_competition_index",
 "type": "continuous",
 "unit": "index",
 "range": [1.28, 4.85],
 "missing_rate": 0.0064,
 "transformation": "log"
 },
 {
 "name": "customer_satisfaction",
 "type": "continuous",
 "unit": "score_1_10",
 "range": [4.85, 9.67],
 "missing_rate": 0.125,
 "transformation": "none"
 },
 {
 "name": "operational_cost_ratio",
 "type": "continuous",
 "unit": "percentage",
 "range": [0.142, 0.387],
 "missing_rate": 0.0272,
 "transformation": "logit"
 },
 {
 "name": "technology_adoption_rate",
 "type": "continuous",
 "unit": "percentage",
 "range": [0.089, 0.678],
 "missing_rate": 0.0713,
 "transformation": "logit"
 },
 {
 "name": "environmental_investment",
 "type": "continuous",
 "unit": "million_CNY",
 "range": [28.4, 298.5],
 "missing_rate": 0.0,
 "transformation": "log"
 },
 {
 "name": "policy_implementation_time",
 "type": "continuous",
 "unit": "months",
 "range": [3.0, 42.0],
 "missing_rate": 0.0,
 "transformation": "none"
 },
 {
 "name": "regulatory_stringency",
 "type": "continuous",
 "unit": "index_1_10",
 "range": [1.50, 7.00],
 "missing_rate": 0.0,
 "transformation": "none"
 },
 {
 "name": "stakeholder_engagement",
 "type": "continuous",
 "unit": "score_1_10",
 "range": [3.20, 9.80],
 "missing_rate": 0.0,
 "transformation": "none"
 },
 {
 "name": "system_reliability_index",
 "type": "continuous",
 "unit": "proportion",
 "range": [0.867, 0.998],
 "missing_rate": 0.0,
 "transformation": "logit"
 }
 ],
 "target_variable": {
 "name": "green_transformation_effect",
 "type": "continuous",
 "unit": "composite_index",
 "range": [0.0, 1.0],
 "computation_method": "weighted_average_of_indicators"
 }
}

# Supplementary Appendix H: Posterior Predictive Check Results

## Table S15. Posterior Predictive Check Summary Statistics

| Statistic | Observed Value | Simulated Mean | Simulated SD | Bayesian p-value |
| --- | --- | --- | --- | --- |
| Mean | 0.542 | 0.538 | 0.024 | 0.45 |
| Standard Deviation | 0.127 | 0.131 | 0.018 | 0.38 |
| Skewness | 0.234 | 0.198 | 0.087 | 0.29 |
| Kurtosis | 2.876 | 2.923 | 0.156 | 0.62 |
| Minimum | 0.285 | 0.278 | 0.034 | 0.41 |
| Maximum | 0.912 | 0.924 | 0.042 | 0.34 |
| 25th Percentile | 0.456 | 0.449 | 0.028 | 0.39 |
| 75th Percentile | 0.634 | 0.641 | 0.031 | 0.56 |
| Autocorrelation (Lag 1) | 0.723 | 0.698 | 0.045 | 0.28 |
| Autocorrelation (Lag 4) | 0.456 | 0.478 | 0.067 | 0.63 |

Note: Bayesian p-values between 0.05 and 0.95 indicate acceptable model fit. Values outside this range suggest potential model misspecification.

## Table S16. Comparison of Variational Inference vs. MCMC Estimates

| Parameter | VI Posterior Mean | VI Posterior SD | MCMC Posterior Mean | MCMC Posterior SD | Relative Difference (Mean) | Relative Difference (SD) |
| --- | --- | --- | --- | --- | --- | --- |
| Renewable Energy Effect | 0.282 | 0.034 | 0.289 | 0.042 | 2.4% | 19.0% |
| Carbon Pricing Effect | 0.234 | 0.029 | 0.241 | 0.035 | 2.9% | 17.1% |
| Regulatory Effect | 0.190 | 0.025 | 0.195 | 0.031 | 2.6% | 19.4% |
| Efficiency Standards Effect | 0.143 | 0.022 | 0.148 | 0.027 | 3.4% | 18.5% |
| Intercept | 0.285 | 0.018 | 0.291 | 0.024 | 2.1% | 25.0% |
| Noise Variance | 0.0089 | 0.0012 | 0.0092 | 0.0016 | 3.3% | 25.0% |

Note: MCMC estimates based on Hamiltonian Monte Carlo with 4 chains, 2000 samples each (1000 warmup). Relative difference calculated as (MCMC - VI) / MCMC.

# Supplementary Appendix I: Processed Dataset Sample

## Table S17. Sample Data Records (First 20 Observations)

| ID | Year | Quarter | Prefecture | Carbon_Intensity | Renewable_Share | Policy_Intensity | Efficiency_Rating | Green_Investment | Compliance_Score | Effect |
| --- | --- | --- | --- | --- | --- | --- | --- | --- | --- | --- |
| 1 | 2018 | Q1 | Fuzhou | 0.623 | 0.198 | 2.34 | 2.45 | 0.089 | 72.3 | 0.312 |
| 2 | 2018 | Q1 | Xiamen | 0.587 | 0.234 | 2.67 | 2.78 | 0.112 | 78.5 | 0.378 |
| 3 | 2018 | Q1 | Quanzhou | 0.645 | 0.178 | 2.12 | 2.34 | 0.078 | 68.9 | 0.289 |
| 4 | 2018 | Q1 | Zhangzhou | 0.678 | 0.156 | 1.89 | 2.12 | 0.067 | 64.2 | 0.256 |
| 5 | 2018 | Q1 | Nanping | 0.712 | 0.145 | 1.67 | 1.98 | 0.056 | 58.7 | 0.223 |
| 6 | 2018 | Q2 | Fuzhou | 0.612 | 0.212 | 2.56 | 2.56 | 0.098 | 74.5 | 0.334 |
| 7 | 2018 | Q2 | Xiamen | 0.578 | 0.245 | 2.89 | 2.89 | 0.123 | 80.2 | 0.401 |
| 8 | 2018 | Q2 | Quanzhou | 0.634 | 0.189 | 2.34 | 2.45 | 0.087 | 71.2 | 0.312 |
| 9 | 2018 | Q2 | Zhangzhou | 0.667 | 0.167 | 2.12 | 2.23 | 0.076 | 66.8 | 0.278 |
| 10 | 2018 | Q2 | Nanping | 0.698 | 0.156 | 1.89 | 2.09 | 0.065 | 61.4 | 0.245 |
| 11 | 2018 | Q3 | Fuzhou | 0.598 | 0.223 | 2.78 | 2.67 | 0.108 | 76.8 | 0.356 |
| 12 | 2018 | Q3 | Xiamen | 0.567 | 0.256 | 3.12 | 2.98 | 0.134 | 82.1 | 0.423 |
| 13 | 2018 | Q3 | Quanzhou | 0.623 | 0.198 | 2.56 | 2.56 | 0.095 | 73.4 | 0.334 |
| 14 | 2018 | Q3 | Zhangzhou | 0.656 | 0.178 | 2.34 | 2.34 | 0.084 | 68.9 | 0.298 |
| 15 | 2018 | Q3 | Nanping | 0.687 | 0.165 | 2.12 | 2.18 | 0.073 | 63.7 | 0.265 |
| 16 | 2018 | Q4 | Fuzhou | 0.587 | 0.234 | 3.01 | 2.78 | 0.118 | 78.9 | 0.378 |
| 17 | 2018 | Q4 | Xiamen | 0.556 | 0.267 | 3.34 | 3.09 | 0.145 | 83.6 | 0.445 |
| 18 | 2018 | Q4 | Quanzhou | 0.612 | 0.209 | 2.78 | 2.67 | 0.104 | 75.6 | 0.356 |
| 19 | 2018 | Q4 | Zhangzhou | 0.645 | 0.189 | 2.56 | 2.45 | 0.093 | 71.2 | 0.318 |
| 20 | 2018 | Q4 | Nanping | 0.676 | 0.176 | 2.34 | 2.28 | 0.082 | 65.8 | 0.285 |

# End of Supplementary Materials
